# Metal-dependent enzyme symmetry guides the biosynthetic flux of terpene precursors

**DOI:** 10.1038/s41557-023-01235-9

**Published:** 2023-06-12

**Authors:** Felix Ecker, Abith Vattekkatte, Wilhelm Boland, Michael Groll

**Affiliations:** 1 Center for Protein Assemblies, Technical University of Munich, Garching, Germany; 2 Department of Bioorganic Chemistry, Max Planck Institute for Chemical Ecology, Jena, Germany

**Keywords:** Enzyme mechanisms, X-ray crystallography, Metals

## Abstract

Terpenoids account for more than 60% of all natural products, and their carbon skeletons originate from common isoprenoid units of different lengths such as geranyl pyrophosphate and farnesyl pyrophosphate. Here we characterize a metal-dependent, bifunctional isoprenyl diphosphate synthase from the leaf beetle *Phaedon cochleariae* by structural and functional analyses. Inter- and intramolecular cooperative effects in the homodimer strongly depend on the provided metal ions and regulate the biosynthetic flux of terpene precursors to either biological defence or physiological development. Strikingly, a unique chain length determination domain adapts to form geranyl or farnesyl pyrophosphate by altering enzyme symmetry and ligand affinity between both subunits. In addition, we identify an allosteric geranyl-pyrophosphate-specific binding site that shares similarity with end-product inhibition in human farnesyl pyrophosphate synthase. Our combined findings elucidate a deeply intertwined reaction mechanism in the *P. cochleariae* isoprenyl diphosphate synthase that integrates substrate, product and metal-ion concentrations to harness its dynamic potential.

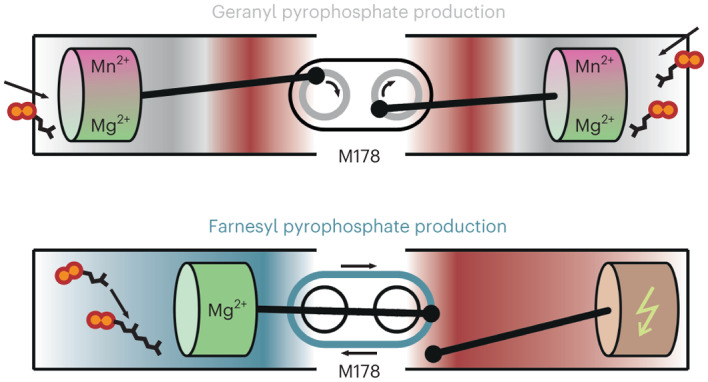

## Abstract (web summary)

The metal-dependent, bifunctional isoprenyl diphosphate synthase PcIDS1 from the leaf beetle *Phaedon cochleariae* integrates substrate, product and metal-ion concentrations to tune its dynamic reactivity. Now structural and functional analyses reveal that this enzyme uses both catalytic centres to form geranyl pyrophosphate, while one domain is inactivated during farnesyl pyrophosphate production.

## Main

**Fig. 1:**
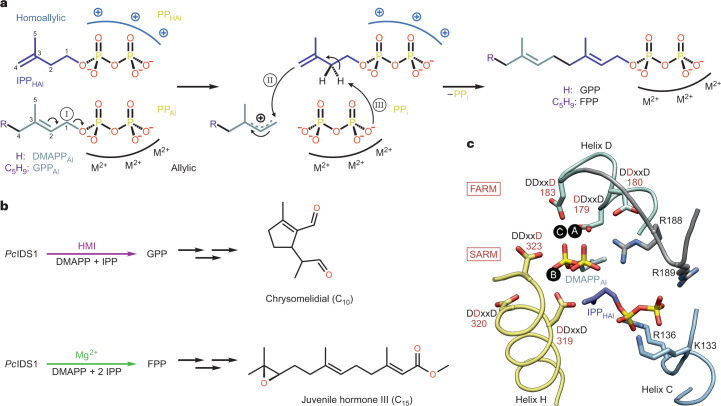
Reaction mechanism and substrate coordination in scIDS. **a**, Catalysis is uniform in this class of enzymes and starts with a prenyl substrate (DMAPP or GPP) bound to the allylic site (Al). A trinuclear, divalent metal cluster surrounds the pyrophosphate moiety (PP_Al_) and facilitates ionization of the ester bond (I). Basic residues of the homoallylic site (HAl) bind IPP, which attacks the nascent carbocation at C_1_ of the prenyl moiety (II). The generated inorganic pyrophosphate (PP_i_) deprotonates at C_2_ (III) of the intermediate and restores the allylic character of the elongated metabolite (GPP or FPP). **b**, In *P. cochleariae*, GPP is the main product of PcIDS1 in the presence of HMIs and precursor of the defensive monoterpene (C_10_) chrysomelidial. FPP formation is driven by Mg^2+^ and essential for sesquiterpene hormones (C_15_) during larval development. **c**, Active site architecture of scIDS enzymes shown for PcIDS1 in complex with DMAPP_Al_ and IPP_HAl_. The first and second aspartate-rich DDxxD motifs (FARM and SARM; D, aspartate; x, any amino acid; red numbers indicate residue position) on helix D and helix H coordinate Mg^2+^ at positions A, B and C. Colour coding is described in the Methods.

Terpenes and terpenoids form the largest group of natural products, with currently over 80,000 known members, of which many display intriguing pharmaceutical properties^1^. Despite being chemically diverse agents of intraspecies and interspecies communication, as well as cellular integrity and proliferation, they emerge from only two isomeric building blocks, dimethylallyl pyrophosphate (DMAPP, C_5_) and isopentenyl pyrophosphate (IPP, C_5_)^2^. Short chain isoprenyl diphosphate synthases (scIDSs) elongate DMAPP to geranyl pyrophosphate (GPP, C_10_) and further to farnesyl pyrophosphate (FPP, C_15_) by consuming IPP in each step^3^. The uniform mechanism of this enzyme class requires three divalent cations that coordinate substrates at an allylic binding pocket (Al site) between the first and second aspartate-rich motif (FARM and SARM). The metal cluster provides an ionizing force that facilitates the dissociation of the pyrophosphate moiety (PP_Al_) once IPP binds to basic residues at the proximal homoallylic site (HAl; Fig. 1). The terminal double bond of IPP_HAl_ attacks the nascent carbocation and connects the C_5_ precursor with the prenyl unit. Ultimately, the released inorganic pyrophosphate (PP_i_) deprotonates the extended metabolite at C_2_ and forms the *trans* double bond. If the chain length determination domain of the enzyme provides access to the generated allylic product, the elongation reaction repeats^4^.

FPP synthases (FPPSs) are distributed in all three kingdoms of life and catalyse both GPP and FPP formation, with the latter being the preferred reaction under physiological conditions. By contrast, GPP-specific synthases (GPPSs) are predominantly found in plants, but some insects, especially aphids and beetles, use GPP to yield defensive and pheromonal monoterpenes^5–7^. To date, only a single animal GPPS has been identified, in a bark beetle^8^, suggesting that alternative regulatory mechanisms to accumulate GPP exist within the repertoire of FPPS. Indeed, several scIDSs in insects control the biosynthesis of mono- (C_10_) and sesquiterpenes (C_15_) to meet the organism’s current needs^9–12^. Isoprenyl diphosphate synthase in *P. cochleariae* (PcIDS1, EC 2.5.1.1) produces FPP in the presence of Mg^2+^, but preferably forms GPP when exposed to the heavy metal ions (HMIs) Co^2+^ or Mn^2+^. Using C_10_ units as a building block, this leaf beetle secretes monoterpenes like chrysomeldial to protect itself against predation in its larval stadium. However, insects constantly require sesquiterpenoid hormones that drive large transformations during metamorphosis (Fig. 1b). Thus, balancing the concentrations of terpene precursors in accordance with environmental and developmental demands is an essential cellular process^13^.

Here we elucidate catalytic principles in PcIDS1 that either break or enforce dimer symmetry in response to HMIs. High-resolution crystal structures combined with functional and mutagenetic analyses reveal how different conformational states determine the product spectrum. In addition, a Mg^2+^–water cluster was identified that regulates the elongation reaction by reducing the affinity for IPP. We demonstrate that Mg^2+^ deactivates one of both active sites in PcIDS1 during FPP formation, whereas HMIs recruit GPP to a specific, allosteric pocket similar to end-product inhibition in the human FPP synthase^14^. Overall, our combined findings provide insights into the biosynthetic flux of terpene precursors towards biological defence or physiological development.

## Results

**Fig. 2:**
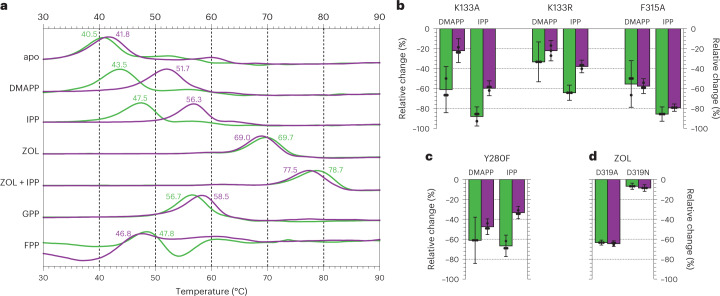
Thermal stability of PcIDS1:metal:ligand complexes. **a**, The fluorescence signal of recombinant PcIDS1 (6.25 µM) was recorded between 30 and 90 °C in the absence of (apo) and together with 200 µM of respective ligands. The graph displays the time-based derivative in relative units. The mean melting temperature is annotated in degrees Celsius next to the curves observed in MgCl_2_ buffer alone (5 mM, green) and with MnCl_2_ (1 mM, purple). **b**,**c**, Relative changes in thermal stability (compared to wild-type protein) for different protein mutants in the presence of DMAPP and IPP. **d**, ZOL binding stability in variants of the metal-coordinating SARM residue D319. Bars indicate the average value of three measurements (shown as dots); whiskers represent the relative error of the mean between mutant and wild-type protein. A detailed data analysis is in Supplementary Fig. 2.

Compared to well-studied scIDS enzymes that specifically produce C_10_, C_15_ or C_20_ isoprene precursors, the product chain length of PcIDS1 depends heavily on the provided metal cofactor. This synthase yields 96% GPP and only 4% FPP in the presence of Mn^2+^ or Co^2+^, whereas 18% GPP and 82% FPP form with Mg^2+^ (ref. ^10^). To elucidate the HMI-dependent molecular processes that suppress FPP accumulation, PcIDS1 was recombinantly expressed and purified (Supplementary Fig. 1). Protein stability was optimal in MgCl_2_ buffers and tolerated the addition of MnCl_2_, while CoCl_2_ facilitated precipitation. Next, we characterized the denaturation temperatures of different PcIDS1:metal:ligand complexes by thermal shift assays (Fig. 2a and Supplementary Fig. 2). In the absence of ligands, the stability of wild-type protein (41 °C) was not affected by Mn^2+^. For the allylic substrate DMAPP, the temperature shift in the presence of Mn^2+^ (Δ10 °C) was much stronger compared to Mg^2+^ (Δ3 °C). This discrepancy correlates with the higher affinity of PcIDS1 for DMAPP with Mn^2+^ (ref. ^10^), which might allow DMAPP to occupy the HAl site at high concentrations^15^. A similar metal dependency was observed with IPP (Δ14 °C for Mn^2+^, Δ7 °C for Mg^2+^), further highlighting the preference of HMIs to coordinate C_5_ substrates. The significant change in thermal stability with either metal suggests that IPP coordinates the HAl as well as the Al site in the absence of competing ligands. However, Mn^2+^ had no effect on the drastically increased protein stability (Δ28 °C) with the Al-specific ligand zoledronic acid (ZOL; ref. ^16^). The denaturation temperature, however, rose further when IPP was added (Δ37 °C), supposing that the inhibitor and substrate cooperatively interact with the Al site as well as the HAl site. For GPP, the product of the first elongation, the distinct thermal shift (Δ17 °C) was indifferent to the metal species, and FPP only had a moderate influence on protein stability (Δ5 °C for Mn^2+^, Δ7 °C for Mg^2+^). Therefore, our thermal shift assay results provide valuable information on ligand affinity to elucidate metal-dependent processes in PcIDS1 at the molecular level.

### Catalytic principles of PcIDS1

**Extended Data Fig. 1:**
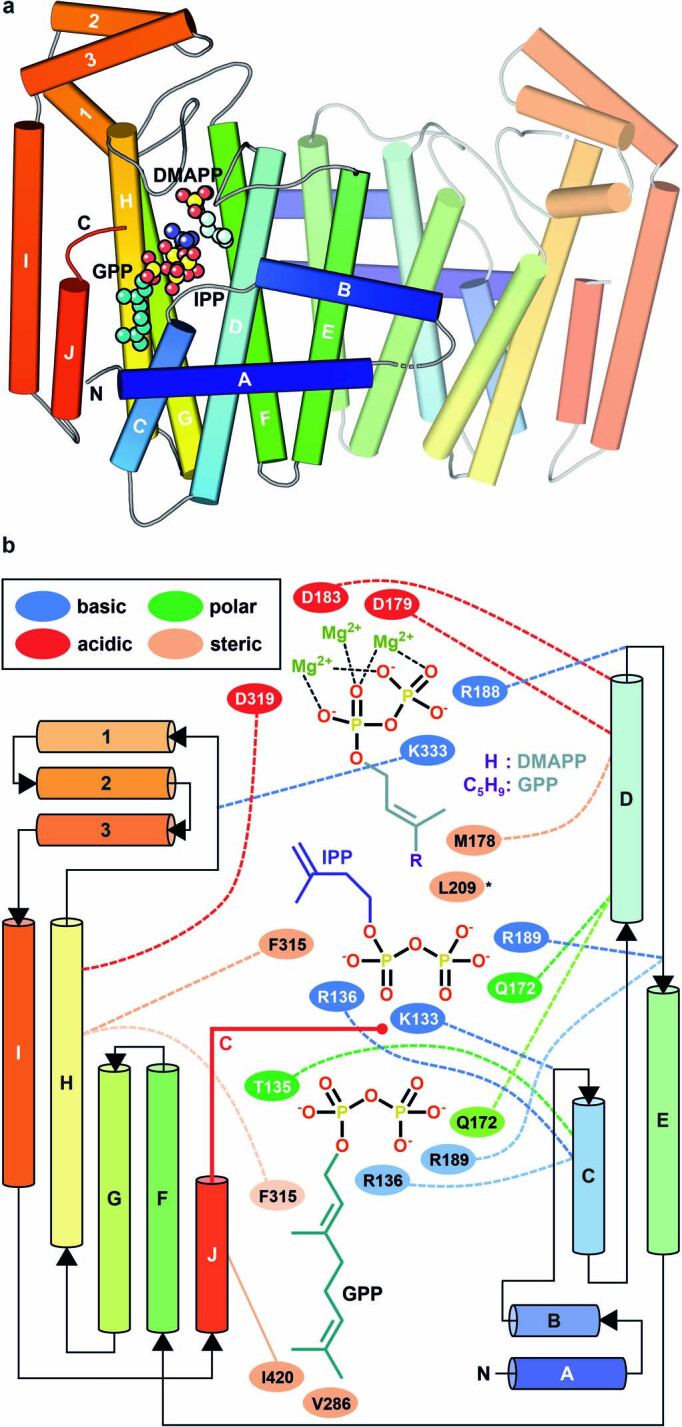
Topology of *Pc*IDS1. **a**, Cartoon representation of *Pc*IDS1 with α-helices shown as cylinders in a spectrum from blue to red (N- to C-terminus). Helices are labelled from A-H, 1-3 and I-J. Sticks and balls representations of the natural ligands highlight their respective binding mode. **b**, Topology of the arrangement in a single subunit. The C_α_ chain trace is indicated by arrows. Residues coordinating ligands in the allylic (Al, cyan), homoallylic (HAl, purple) and allosteric (Allo, teal) site are connected to their respective position in the enzyme and coloured according to the nature of the interaction. An asterisk indicates a residue from the adjacent subunit. The C-terminus that orders upon binding of IPP_HAl_ or GPP_Allo_ is highlighted in red.

To obtain molecular insights into PcIDS1, we first determined the apo crystal structure in the presence of Mg^2+^ (Protein Data Bank (PDB) 8A6U). The enzyme adopts the conserved scIDS topology (best distance matrix alignment (DALI) hit^17^, PDB 3EWG, FPPS from *Trypanosoma brucei*^18^, Z-score (similarity of equivalent Cα-Cα distances among two proteins) 36.1, 30% sequence identity) with a dimer interface of 2,000 Å^2^ (ref. ^19^). Each subunit (sub_A_ and sub_B_) consists of 13 helices (αA to αJ and α1 to α3; Extended Data Fig. 1a). In both chains, the N terminus and C terminus as well as the loops between αD–αE (183–201) and αH–α1 (329–332) are disordered. The FARM region (179–183) is partially resolved, while D319 of the SARM (319–323) coordinates a pentahydrated Mg^2+^ at site B (Mg^2+^_B_).

**Fig. 3:**
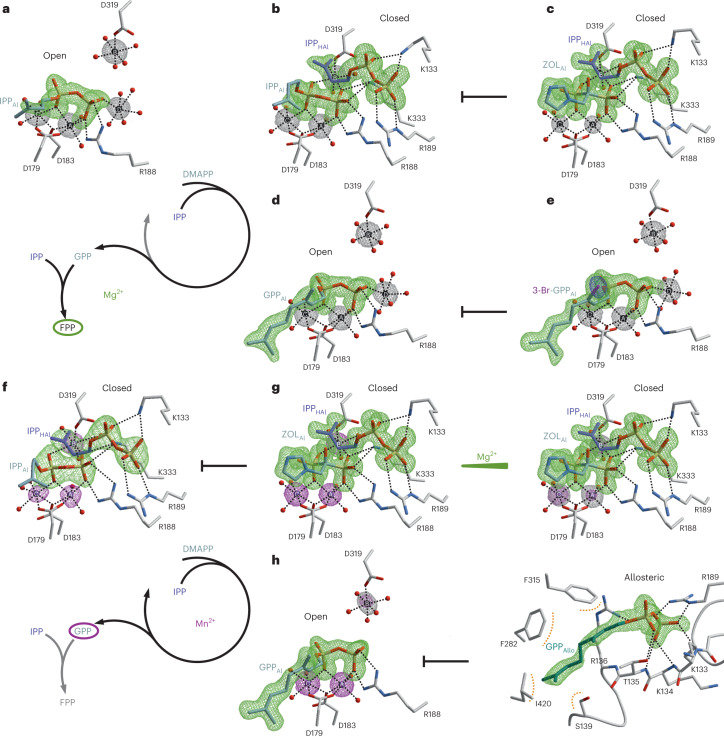
Mg^2+^ ions drive FPP production in PcIDS1. Snapshots of the active site during catalysis and in complex with inhibitors. Metal ions are drawn in black (Mg^2+^) and pink (Mn^2+^), H-bonds are illustrated as black dots. Omit electron density maps are shown in green (F_O_–F_C_, 3.0 σ), grey (2F_O_–F_C_, 1.0 σ), pink (ano Mn, 10.0 σ) and blue (ano Br, 10.0 σ). **a**, PcIDS1_Mg_:IPP (PDB 8A6V); IPP imitates the native DMAPP_Al_ moiety with a fourth Mg^2+^ (site D) coordinating P_β_. **b**, PcIDS1_Mg_:IPP:IPP (PDB 8A6V); a second IPP molecule binds to basic residues at the homoallylic site (HAl). Mg^2+^_B_ is pushed towards PP_Al_, which adapts an eclipsed conformation to depart as PP_i_. **c**, PcIDS1_Mg_:ZOL:IPP (PDB 8A7C); the bisphosphonate inhibitor ZOL imitates PP_Al_ coordination in the closed state. **d**, PcIDS1_Mg_:GPP (PDB 8A70); upon prenyl elongation and PP_i_ release, GPP binds to the allylic site (Al) in the open state. Site D is occupied by a pentahydrated Mg^2+^ ion. Mg^2+^ drives the repetition of the cycle to form FPP (left). **e**, PcIDS1_Mg_:3-Br-GPP (PDB 8A7A); the bromine atom in 3-Br-GPP prevents carbocation formation. **f**, PcIDS1_Mn_:IPP:IPP (PDB 8A6Z); both subunits form the closed state with IPP_Al_ and IPP_HAl_ with Mn^2+^ at a 100-fold excess of Mg^2+^. **g**, PcIDS1_Mn_:ZOL:IPP; the active site is occupied by Mn^2+^ at sites A through C in complex with the inhibitor ZOL_Al_ and IPP_HAl_ (right, PDB 8A7J). A 50-fold excess of Mg^2+^ reduces the anomalous signal at A and B (left, PDB 8A7K). **h**, PcIDS1_Mn_:GPP:GPP (PDB 8A73); Mn^2+^_A_ and Mn^2+^_C_ sequester the PP_Al_ moiety and prevent coordination of Mg^2+^_D_. A second GPP acts as an allosteric inhibitor (right). GPP is the main product of catalysis in the presence of Mn^2+^ (left). Inhibition arrows are highlighted in black. Colour coding is described in the Methods. A stereo version of this figure is presented in Extended Data Fig. 3. A comprehensive overview of all structures is shown in Supplementary Figs. 3–6.

**Fig. 4:**
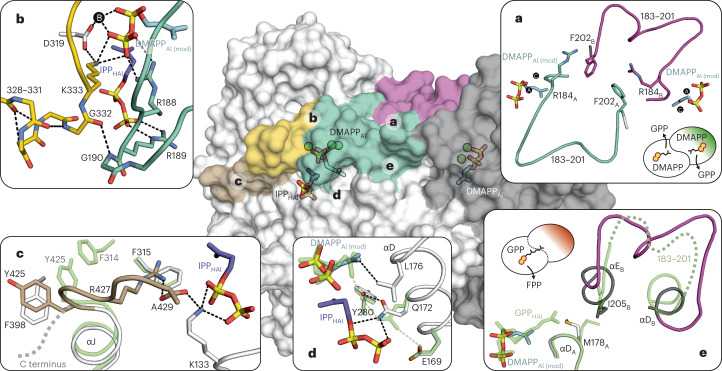
The regulatory framework of PcIDS1. Domains undergoing distinct conformational changes during catalysis are highlighted on a surface representation of PcIDS1_Mg_:IPP:IPP (PDB 8A6V). DMAPP was modelled into the allylic site (DMAPP_Al (mod)_) to chains A (white) and B (grey; Supplementary Fig. 7b). **a**, DMAPP_Al_ induces substrate loop formation (183–201, mint) and initiates a cooperative effect on the second subunit through cation–*π* interactions between R184 and F202 residues (purple). GPP formation can occur in both active sites simultaneously (inset green scheme). **b**, IPP_HAl_ forms the closed state with R188 and R189 (mint) facing the PP moieties. D319 and metal B move towards DMAPP_Al_ and stabilize the eclipsed PP conformation with K333 (gold). Thereby, residues 328–331 arrange into a helix and anchor the substrate loop. **c**, The C terminus (brown) is recruited from a dynamic state (green) by inverting Y425 to engage in *π*-stacking with F398. F315 selects for IPP_HAl_ and provides cation–*π* interactions to R427. The terminal carboxy group of A429 is located between R427 and K133, which stabilizes PP_HAl_. **d**, In the presence of IPP_HAl_, Q172 breaks the hydrogen bond to E169 (green) and displaces Y280, causing a rotation of the DMAPP_Al_ prenyl moiety towards L176. **e**, The C_10_ prenyl chain of GPP disrupts the substrate loop at the N terminus of αE_B_ in the neighbouring subunit by pushing M178_A_ into I205_B_. PcIDS1 is a single-subunit catalyst for FPP production (inset red scheme). Colour coding is described in the Methods.

Next, we determined a Mg^2+^-bound IPP complex (PDB 8A6V) that shows significant discrepancies between the two subunits. Sub_A_ displays an open state and contains a single IPP molecule at the Al site with a 5 Å distance to Mg^2+^_B_, mimicking the native substrate DMAPP (Fig. 3a). D179 and D183 of the FARM rearrange and coordinate PP_Al_ in a staggered conformation by Mg^2+^_A_ and Mg^2+^_C_. Surprisingly, the electron density map depicts an additional pentahydrated metal site (Mg^2+^_D_) at the β-phosphate (P_β_) with a major impact on catalysis (as decribed in the following section). Binding of IPP_Al_ introduces a strong hydrogen bond between R188 and P_β_ that reorders the residues of αD–αE into a defined substrate loop. Notably, R184 and F202 in this region engage in cation–*π* interactions with their equivalents in sub_B_, indicating intersubunit cooperativity between the Al sites (Fig. 4a).

**Extended Data Fig. 2:**
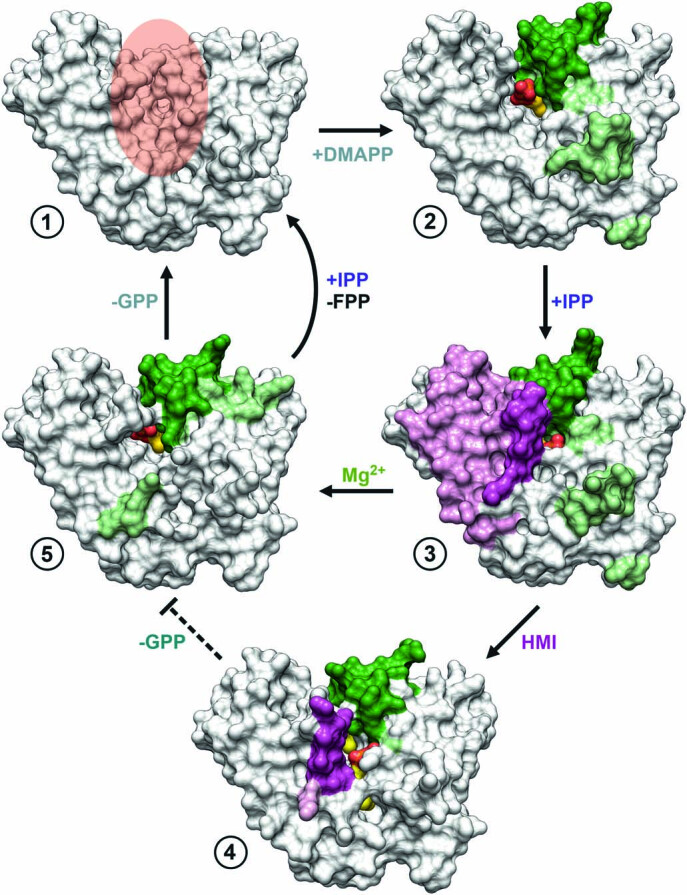
Structural plasticity of *Pc*IDS1 during catalysis. Surface representations of *Pc*IDS1 subunits in complex with different ligands. The apo structure (**1**, PDB ID 8A6U) features a deep central cleft surrounded by disordered regions (highlighted in red). Upon binding of DMAPP_Al_, the substrate loop between αD-E folds around the pyrophosphate (PP) moiety (**2**, PDB ID 8A6V, sub_A_). Areas undergoing significant structural changes are shown in light green and residues disordered in the apo structure in dark green. The binding of IPP_HAl_ (**3**, PDB ID 8A6V, sub_B_) shifts the peripheral domain around the SARM towards the active centre (light pink) and orders the region between αH-1 as well as the C-terminus (dark pink). The presence of heavy metal ions (HMI) stabilises the accommodation of an allosteric GPP molecule (**4**, PDB ID 8A73) that interferes with IPP_HAl_ binding. The C-terminus adapts a conformation as observed in the closed state. In absence of HMIs or upon release of GPP_Allo_, the *Pc*IDS1:GPP complex (**5**, PDB ID 8A70) can either form FPP or start a new reaction cycle. Colour coding is described in *Materials and Methods*.

However, sub_B_ forms the closed state with two IPP molecules and depicts extensive conformational changes compared to the open state (Fig. 3b and Supplementary Table 4). Here, the domain around the SARM is rearranged and pushed towards the Al site together with α3 and the N terminus of αI (Extended Data Fig. 2). The flexible region 327–336 forms a short α-helix that anchors K333 between PP_Al_ and D319 (Fig. 4b). This induced fit moves Mg^2+^_B_ by 4 Å to interact with both phosphates of IPP_Al_, while Mg^2+^_D_ is released. Thereby, the PP_Al_ moiety is forced into an eclipsed configuration that promotes carbocation formation. Binding of the second IPP molecule to K133, R136 and R189 at the homoallylic site recruits the C terminus (425–429; Extended Data Fig. 1b). Stabilized by cation–*π* interactions with R427, the F315 side chain positions IPP_HAl_ for nucleophilic attack (Fig. 4c). These structural observations are supported by a decreased thermal resilience of the K133R, K133A and F315A variants in complex with DMAPP and IPP (Fig. 2b).

The comparison between the open (sub_A_) and closed (sub_B_) states reveals a feedback relation between the HAl and Al sites. Binding of IPP_HAl_ displaces interactions between E169, Q172 and Y280, resulting in a 120° clockwise rotation of the allylic prenyl unit (Fig. 4d and Supplementary Fig. 7c). The significantly reduced stability for DMAPP and IPP in the Y280F mutant indicates catalytic relevance of this regulatory cascade (Fig. 2c). Modelling of DMAPP_Al_ and IPP_HAl_ into the active site shows that reorientation of the allylic substrate results in an ideal distance (3.2 Å) between its C_1_ atom and the homoallylic double bond, facilitating C–C bond formation.

To investigate the second elongation step from GPP to FPP, we determined the complex structure of PcIDS1_Mg_:GPP (PDB 8A70). In the presence of Mg^2+^, GPP_Al_ binds to the metal cluster (A–D) at the Al site in sub_A_ (open state; Fig. 3d) and sub_B_ remains vacant. While GPP_Al_ adopts the binding mode of IPP_Al_ with C_1_–C_5_, the distal C_6_–C_10_ unit inverts the M178 side chain and generates a spacious specificity pocket that is inaccessible in the IPP_Al_ complex. To elucidate the structural features of FPP formation in the closed state, we applied IPP together with a geranyl pyrophosphate derivative brominated at position C3 (3-Br-GPP). In this surrogate, the electrophilic bromine prevents carbocation formation and blocks catalysis^20^. Our crystallographic studies show that 3-Br-GPP mimics GPP_Al_ coordination, and the halogen atom occupies a similar position as the native 3-methyl group (Fig. 3e; PDB 8A7A). However, the HAl site remains vacant as 3-Br-GPP competes with IPP_HAl_ binding by stabilizing the open state through a polar interaction between Y280 and Br (Supplementary Fig. 4f). In the absence of experimental data, we modelled the active GPP_Al_:IPP_HAl_ complex (Supplementary Fig. 7d). To reach the closed state, GPP_Al_ must undergo a similar reorientation as that of DMAPP_Al_, suggesting that GPP and FPP syntheses follow the same catalytic process. Yet, the relationship between structure and product distribution remains unresolved.

### Dimer symmetry governs product distribution

**Extended Data Fig. 4:**
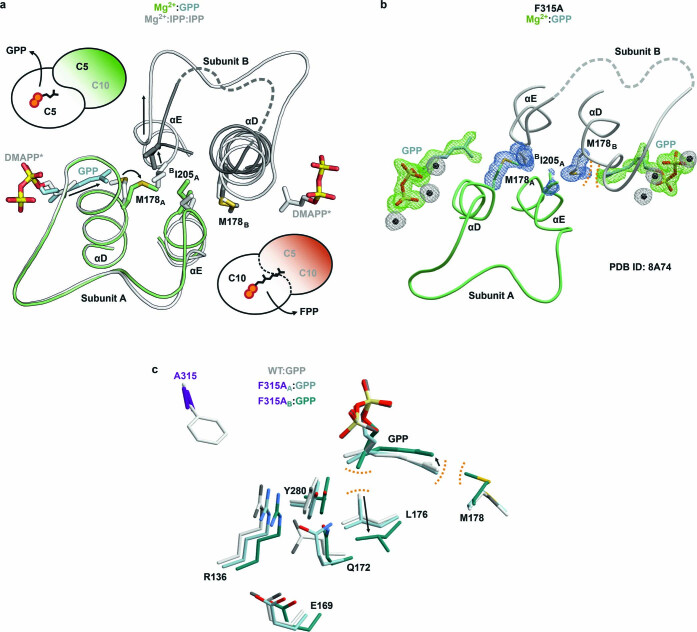
GPP_Al_-binding disrupts the substrate loop and HAl site in the neighbouring subunit. **a**, Superposition of *Pc*IDS1_Mg_:GPP (coloured) and *Pc*IDS1_Mg_:IPP:IPP (white, DMAPP* in the figure): The C_10_ prenyl chain of GPP_Al_ disrupts substrate loop formation in the neighbouring subunit by displacing M178_A_ and I205_B_, which disorders the N-terminus of αE_B_. While GPP synthesis can take place synchronistically (top left scheme), the reaction to FPP can only occur successively (bottom right). **b**, *Pc*IDS1_F315A_Mg_:GPP: In presence of GPP_Al_ in sub_A_ (green), IPP_HAl_ coordination is prevented in sub_B_ (grey). The FARM at the disrupted substrate loop retains affinity for allylic ligands. GPP_Al_ adopts a non-physiological, kinked conformation in sub_B_ to avoid steric clashes (orange dashes) with M178. **c**, Superposition of *Pc*IDS1_Mg_:GPP (grey) with sub_A_ and sub_B_ of *Pc*IDS1_F315A_Mg_:GPP (cyan and teal): In absence of the phenyl sidechain, major rearrangements are observed for the regulatory network between R136, E169, Q172 and Y280. The kinked conformation of the GPP_Al_ moiety in F315A_B displaces L176 with its 3-methyl group (arrows). However, C_5_ ligands would not interfere with M178 and L176 and could still bind to sub_B_ in presence of GPP_Al_. Colour coding is described in *Materials and Methods*.

Structural analysis of PcIDS1_Mg_:GPP (PDB 8A70) reveals an asymmetric complex in which the ligand is bound only to sub_A_. This broken dimer symmetry originates from the C_10_ prenyl moiety that flips the side chain of M178 in sub_A_ and displaces I205 in the adjacent sub_B_ (Fig. 4e). The rearrangements disrupt the substrate loop and destabilize the Al and HAl sites of sub_B_ (Extended Data Fig. 4a). Interestingly, the structure of the F315A variant reveals that GPP_Al_ still binds to both subunits in response to a modified catalytic network (PDB 8A74; Extended Data Fig. 4b). While GPP_Al_ follows the described binding mode in sub_A_, the orientation of M178 in sub_B_ forces the prenyl moiety of the ligand into a kinked conformation. This unphysiological state is stabilized by the metal cluster, and the substrate loop in sub_B_ is only defined at the FARM region. Although this asymmetric complex displays an affinity for ligands at the Al site, IPP_HAl_ cannot coordinate to sub_B_ unless GPP_Al_ dissociates from sub_A_. In consequence, PcIDS1 acts as a single-subunit catalyst during FPP formation.

On the other hand, the PcIDS1_Mg_ complex with IPP_Al_ (PDB 8A6V) displays intersubunit cooperativity between the two substrate loops. However, IPP_HAl_ is bound only to one subunit and does not prove that catalysis can take place simultaneously in both active sites. To obtain a symmetric complex with IPP_HAl_ in both subunits, a strong ligand for the Al site like ZOL is required. This inhibitor significantly increases the thermal stability of all PcIDS1 variants apart from D319A, which prevents coordination of Mg^2+^_B_ (Fig. 2 and Supplementary Figs. 2 and 4h). These results indicate that the PcIDS1:ZOL complex favours the closed state and induces formation of the HAl site, which is confirmed by the 9 °C shift upon addition of IPP. Indeed, the structure of PcIDS1_Mg_:ZOL:IPP (PDB 8A7C) displays symmetry between sub_A_ and sub_B_ and mimics the active site prior to GPP synthesis (Fig. 3c). The protonated imidazole group of ZOL_Al_ imitates the nascent carbocation of DMAPP_Al_, while the central hydroxymethyl group constrains the bisphosphonate to a fixed conformation. With four negative charges, bisphosphonate resembles the eclipsed PP_Al_ moiety and engages with all metal sites to induce IPP_HAl_ binding in its native orientation (Supplementary Fig. 5b). Thus, the binding strength of C_5_ ligands at the Al site correlates with the affinity for IPP_HAl_ and promotes catalysis in both subunits.

### Mn^2+^ ions increase affinity for the allylic ligand

To determine the binding mode of HMIs, we solved the crystal structure of PcIDS1_Mn_:ZOL:IPP in a buffer containing a mixture of MgCl_2_ and MnCl_2_ (5:1 stoichiometry; PDB 8A7J). The subunits are identical to PcIDS1_Mg_:ZOL:IPP (PDB 8A7C) apart from the anomalous Mn signal for all three metal positions (Fig. 3g, left and Supplementary Table 4). The influence of environmental HMI concentrations was evaluated by a second structure in the presence of 50-fold Mg^2+^ excess over Mn^2+^ (PDB 8A7K). Despite the abundance of Mg^2+^, sites A and B still harbour Mn^2+^ (Fig. 3g, right), confirming the preference of the enzyme for ligands coordinated by HMIs. Unlike Mn^2+^_A_ and Mn^2+^_B_, which rest in a rigid, bidentate coordination with the phosphonate groups of ZOL_Al_, position C can be selected through interactions with the FARM during substrate binding. Accordingly, Mn^2+^_C_ remains the dominant metal species even at high Mg^2+^ concentrations to enhance the ionization of P_α_ during catalysis and stabilize the nascent carbocation.

However, the enforced closed state through ZOL_Al_ coordination does not provide information about the metal-dependent interplay between the Al and HAl sites. Therefore, we applied IPP as a surrogate for DMAPP_Al_ to evaluate Mn^2+^ binding in a native context and solved the structure of PcIDS1_Mn_:IPP:IPP (PDB 8A6Z). The complex forms a symmetric dimer with IPP_Al_ and IPP_HAl_ in the closed state and is similar to sub_B_ of PcIDS1_Mg_:IPP:IPP (PDB 8A6V; Supplementary Fig. 3f). Remarkably, the three metal positions depict a uniform anomalous density for Mn^2+^ despite a 100-fold Mg^2+^ excess in the crystallization buffer (Fig. 3f). Thus, in the absence of IPP_HAl_, the staggered configuration of PP_Al_ selects for Mn^2+^_A_ and Mn^2+^_C_. Once IPP coordinates the HAl site, the high affinity of Mn^2+^ replaces Mg^2+^_B_ and stabilizes the eclipsed orientation of PP_Al_, driving PP_i_ release.

So far, the described Mn^2+^-bound complexes all form symmetric homodimers with ligands at both Al and HAl sites. To determine whether HMIs are also recruited to asymmetric arrangements like those in PcIDS1_Mg_:IPP or PcIDS1_Mg_:GPP, we solved the structure of PcIDS1_Mn_:GPP (PDB 8A73). With both subunits in the open state, GPP_Al_ is bound only to sub_A_. While Mn^2+^_A_ and Mn^2+^_C_ coordinate GPP_Al_, the pentameric water cluster at site B favours Mg^2+^ (Fig. 3h). Interestingly, the complex lacks electron density for a metal at site D, as observed in PcIDS1_Mg_:GPP (PDB 8A70). Apparently, the stronger Lewis acidity of Mn^2+^_A_ and Mn^2+^_C_ reduces the charge density of PP_Al_ and prevents coordination of Mg^2+^_D_. Without this auxiliary metal cluster, P_β_ moves 1.0 Å towards the SARM and interacts with the hydration sphere of Mg^2+^_B_ (Supplementary Fig. 8). These rearrangements align the subunit for IPP_HAl_ coordination and facilitate the transition to the closed state. In consequence, HMIs enhance the intramolecular effect that forms the HAl site when a ligand is bound to Al. This cooperativity stabilizes both HAl sites during GPP synthesis but is limited to sub_A_ upon elongation to FPP. Therefore, HMIs counteract FPP formation by recruiting IPP_HAl_ to sub_B_ and displacing GPP_Al_ from sub_A_.

### Mn^2+^ recruits GPP to an allosteric pocket

The structure of PcIDS1_Mn_:GPP (PDB 8A73) contains well-defined electron density for a second GPP molecule (GPP_Allo_; Fig. 3h) that is absent in the Mg^2+^-bound complex. Its prenyl moiety protrudes into a 7-Å-wide and 15-Å-deep channel between αC and αG as well as αJ in sub_A_. V286 and I420 form the bottom of this specificity pocket and act as a molecular ruler, whereas F282 and F315 engage in *π*-stacking with both allyl groups. Notably, the PP moiety of this allosteric ligand coordinates with residues of the HAl site. While R136 contacts both phosphates, P_β_ is further stabilized by the αC helix dipole and forms a salt bridge with R189 of the substrate loop. GPP_Allo_ keeps the subunit in the open conformation, but the C terminus is directed towards the HAl site, and residues 328–331 form the helical turn as seen in PcIDS1_Mn_:IPP:IPP (PDB 8A6Z). These findings indicate that IPP_HAl_ binding and GPP_Allo_ binding are mutually exclusive, and both depend on the intramolecular effect of HMIs. To elucidate the architecture of the allosteric site in the closed state, we determined the structure of PcIDS1_Mg_:ZOL:GPP (PDB 8A7L). In this complex, ZOL_Al_ displaces GPP_Al_, and the structural rearrangements prevent formation of the allosteric specificity pocket (Supplementary Fig. 6c). However, GPP still binds to the HAl site by inverting its PP moiety and exposing the prenyl unit towards the protein surface. Taken together, GPP_Allo_ represents a competitive inhibitor to IPP_HAl_ that stabilizes the open state in the presence of HMIs.

## Discussion

Our study reveals how PcIDS1 couples metal coordination and dimer symmetry with an allosteric site to fine-tune product distribution during the life cycle of *P. cochleariae*. In this enzyme, diversified regulatory domains evolved from a common scIDS topology to steer the biosynthetic flux of defensive monoterpene or hormonal sesquiterpene precursors. Exogenous metal cofactors shift the product distribution from FPP to GPP. Compared with the abundance of Mg^2+^ in the larval fat body tissue, Mn^2+^ and Co^2+^ are found at a ratio of 1:300 and 1:20,000, respectively^10^. HMIs compensate these low intracellular concentrations with their strong Lewis acidity and engage in stable substrate complexes that bind to PcIDS1 (refs. ^21,22^). Since HMIs accelerate GPP and FPP formation to the same extent, they exploit additional mechanisms to alter product distribution^10,22–25^.

**Fig. 5:**
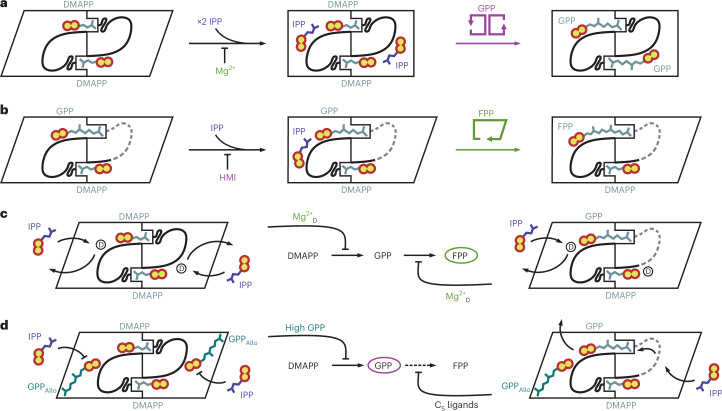
Regulation of PcIDS1 catalysis. The two subunits of the homodimer (sub_A_ and sub_B_) are depicted in either the open (rhombic) or closed (rectangle) state. Substrate loops are drawn in black with unstructured regions as grey dashes. **a**, PcIDS1 catalyses the reaction of DMAPP and IPP to GPP in both subunits at the same time (symmetric, pink scheme). HMIs accelerate the transition to the closed state, while Mg^2+^ slows down catalysis. **b**, The enzyme procedurally converts GPP and IPP to FPP in a single subunit (asymmetric, green scheme). HMIs counteract GPP_Al_ coordination, while Mg^2+^ stabilizes the open state. **c**, Mg^2+^_D_ represents a modulator of IPP_HAl_ binding during GPP (left) and FPP (right) synthesis. **d**, In the presence of HMIs, GPP_Allo_ binds to both substrate loops and prevents GPP accumulation (left). GPP_Allo_ binds only to sub_A_ (right), and C_5_ ligands that coordinate to sub_B_ displace GPP_Al_ from sub_A_ to counteract FPP formation (dashed arrow). An extended reaction network is provided in Supplementary Fig. 11.

Symmetry is a common regulatory principle in scIDS enzymes that use the dimer interface to form selective binding sites for their allylic substrates. While heterodimeric GPPSs with a single active site have been identified^26^, PcIDS1 enters the reaction as a symmetric homodimer. For GPP synthesis, DMAPP_Al_ promotes intersubunit cooperativity and induces structural rearrangements that enable IPP_HAl_ binding. Compared to the apo enzyme, the thermal resilience of PcIDS1:DMAPP is increased, but an even stronger effect is seen for IPP (Fig. 2a). Combined with crystallographic studies, these findings prove a competitive inhibition of IPP_Al_ at high concentrations by binding to the open state as well as the closed state^10,27,28^. In addition, with either IPP_HAl_ or DMAPP_HAl_, HMIs abolish Mg^2+^_D_ coordination at PP_Al_ and enhance GPP formation (Fig. 5a). This metal-dependent cross-talk between the Al and HAl sites results in improved protein stability with both C_5_ ligands and agrees with previous kinetic studies^10^.

Once GPP is formed, it can either dissociate to enter monoterpene biosynthesis or proceed to the Al site for further elongation. Binding of GPP_Al_, however, breaks the dimer symmetry and restricts catalysis to one active site for FPP synthesis (Fig. 5b). Key to this asymmetry is the unique chain length determination domain sequence SLxxM (located at −5 to −1 upstream of the FARM; S, serine; L, leucine; M, methionine; x, any amino acid). So far, characterized scIDSs harbour aromatic sidechains at −5 and −4 that mediate substrate selectivity, and a small residue at −1 (refs. ^4,29–32^). In PcIDS1, S174 of the chain length determination domain motif provides stability at the dimer interface, and L175 forms a hydrophobic patch that guides GPP_Al_ towards M178. An induced flip of the methionine side chain restricts GPP_Al_ to one active site at a time. Such a regulatory connection between distant active sites of homodimeric enzymes was recently described for the human transketolase and represents a fundamental principle of positive or negative cooperativity^33^. Notably, methionine at −1 is conserved in a mosquito scIDS with similar metal-dependent product regulation to PcIDS1 (ref. ^11^).

The binding mode of GPP_Al_ involves significant hydrophobic contacts that stabilize the complex and are independent from the metal cluster at Al. During FPP formation in the presence of Mg^2+^, the additional metal site D, however, decelerates the reaction rate^10^ and selectively recruits IPP_HAl_ only to the catalytic subunit (Fig. 5c). HMIs, on the other hand, put the asymmetric GPP_Al_ complex at a disadvantage by simultaneously increasing the affinity of C_5_ ligands to all Al and HAl sites. In consequence, IPP_HAl_ binding to the disrupted subunit counteracts GPP_Al_ coordination (Fig. 5d). This results in a 20-fold decreased GPP affinity when IPP is applied with HMIs during FPP formation^10^. Thus, HMIs will always prefer the symmetric state with two stabilized substrate loops and accelerate GPP synthesis under physiological conditions.

To prevent GPP accumulation, HMIs fine-tune PcIDS1 activity by generating an allosteric binding site (Fig. 5d). The C_10_ prenyl unit of GPP_Allo_ protrudes into a hydrophobic channel, while the PP_Allo_ moiety competes with IPP_HAl_. This coordination turns the product GPP into an allosteric inhibitor and slows down catalysis by trapping the subunit in a transition state (Supplementary Fig. 9). A similar regulation has been characterized for a C_15_-specific binding site in human FPPS (HsFPPS) that mediates end-product inhibition^14^. However, the human FPP_Allo_ does not interact with the substrate loop and protrudes into a 4-Å-deeper cavity by inverting the prenyl moiety. This orientation is stabilized by L344, whereas the corresponding I420 in PcIDS1 tailors the allosteric binding channel to GPP (Supplementary Fig. 10). When this amino acid is replaced with alanine, the size of the cavity increases, but neither FPP_Allo_, nor GPP_Allo_, nor Mn^2+^ at the Al site are observed in the mutant structure (Supplementary Fig. 6d; PDB 8A7R). These findings highlight that the conserved architecture of allo sites has diverged into distinct roles during evolution. GPP_Allo_ in PcIDS1 is strictly dependent on the allylic metal cluster that induces formation of the HAl site and features a unique mode of action.

Our present study highlights an intricate reaction network for PcIDS1 that combines inter- and intramolecular effects with allosteric regulation. As indicated by previous kinetic assays, HMIs attain a high affinity for DMAPP_Al_ (Michaelis constant (*K*_m_) = 11.6 µM) and IPP_HAl_ (*K*_m_ = 0.84 µM) through cooperative symmetry between the subunits^10^. However, if DMAPP and IPP deplete during GPP accumulation, GPP_Allo_ could promote allosteric feedback and throttle the impact of HMIs on the reaction rate. PcIDS1 catalyses the first elongation step significantly slower with Mg^2+^ alone (25% of maximum velocity of the enzymatic reaction (*v*_max_)) and instead displays higher affinity for GPP_Al_ (*K*_m_ = 1.2 µM)^10^. Our crystal structures highlight that Mg^2+^ stabilizes an asymmetric complex with half-site reactivity to produce FPP, the precursor of sesquiterpene hormones. In vivo, an influx of exogenous HMI cofactors counteracts this second reaction and guides synthesis back to GPP (Supplementary Fig. 11). Thus, the dynamic nature of PcIDS1 allows *P. cochleariae* to fine-tune its terpene metabolism in response to environmental trigger factors during all life stages.

## Methods

### Bacterial culture

The gene of PcIDS1 (GenBank AGE89831.1), which lacks the N-terminal signal peptide (residues 1 to 85)^10^, was cloned into a pET-28b expression vector modified to encode an N-terminal His_6_-SUMO tag. Site-directed mutagenesis (Supplementary Table 1) was performed with the QuikChange II kit (Agilent Technologies) according to the manufacturer’s instructions and verified by Sanger sequencing (GATC Biotech). *Escherichia coli* K12 BL21(DE3) cells were transformed by electroporation and grown in glass shake flasks containing 2 l lysogenic broth (50 mg l^–1^ kanamycin) at 37 °C. After reaching an optical density measured at a wavelength of 600 nm (OD_600_) of 0.6, flasks were stored at 4 °C for 30 minutes before adding 1 mM isopropylthiogalactoside (final concentration) to induce protein expression, which occurred overnight at 20 °C. Cell pellets were collected by centrifugation, washed once with 0.9% (w/v) NaCl and stored at −20 °C.

### Protein purification

*E. coli* pellets of 10 g were dissolved in 50 ml buffer A (100 mM Tris/HCl pH 7.5, 500 mM NaCl, 10% (v/v) glycerol, 20 mM imidazole, 10 mM 2-mercaptoethanol (β-ME), 5 mM MgCl_2_) and solubilized by sonication (Branson Digital Sonifier 250). After centrifugation (40,000*g*, 4 °C, 30 min), the supernatant was applied (5 ml min^−1^) to a 5 ml HisTrap HP column with an ÄKTA Pure system (GE Healthcare), previously equilibrated with buffer A. Buffer A containing 5% buffer B (buffer A with 500 mM imidazole) was used to wash the column until the absorbance signal (280 nm) returned to the baseline. The protein of interest was eluted by a linear gradient (5–100% buffer B) within 50 ml total volume. Fractions containing sufficient concentrations of pure protein were pooled and spiked with 0.5 mg SUMO protease (Ulp1 from *Saccharomyces cerevisiae*) and dialysed overnight at 4 °C against 5 l buffer C (20 mM Tris/HCl pH 7.5, 100 mM NaCl, 10% (v/v) glycerol, 1 mM β-ME, 5 mM MgCl_2_). HisTrap affinity chromatography was repeated, and the flow-through was concentrated to 2 ml using Amicon Ultra-15 centrifugal filters. Centrifugation (20,000*g*, 4 °C, 10 min) removed residual protein aggregates, and the supernatant was used for size exclusion chromatography with a HiLoad Superdex 200 16/60 column in buffer D (buffer C containing 1 mM tris(2-carboxyethyl)phosphine (TCEP) instead of β-ME) at 1.5 ml min^−1^. High purity peak fractions were pooled, concentrated to at least 40 mg ml^−1^ (1 mM) and either used immediately or stored at −80 °C.

### Thermal shift assay

A 96-well transparent polymerase chain reaction plate was prepared with 17 µl buffer in each well (final concentrations, 100 mM MES buffer pH 7.0, 100 mM NaCl, 10% (v/v) glycerol, 5 mM MgCl_2_, 2.5 mM TCEP) both with and without addition of 1 mM MnCl_2_. Ligand molecules (in 1 µl H_2_O) were added to achieve final concentrations of 25 and 200 µM, respectively. In the presence of ZOL, IPP and GPP were applied at 100 µM. Some 1 µl of a 0.25 mg ml^−1^ protein–buffer solution (6.25 µM final concentration) was added using a Phoenix nano-dispenser system (Art Robbins Instruments), followed by 1 µl of an aqueous 1:40 dilution of Sypro Orange Stain (Merck). Plates were sealed, carefully shaken and centrifuged (1,000*g*, 4 °C, 5 min) to remove air bubbles. All samples were equilibrated to 4 °C in a CFX96 reverse transcriptase-polymerase chain reaction (RT-PCR) system (Biorad), and emission was detected while the temperature increased by 0.5 °C in 10 s intervals to 95 °C. Melting temperatures were derived from the inflection points of the fluorescence signal with CFX Maestro (v.4.1) and analysed (in triplicate) with OriginPro 2020 (v.9.7.0.185).

### Protein crystallization and structure determination

Crystals of PcIDS1 wild-type and mutant proteins were grown using sitting drop vapour diffusion at 20 °C. Protein samples were applied at 20 mg ml^–1^ in buffer D (with/without 1 mM MnCl_2_) and mixed with reservoir solution by an Oryx4 system (Douglas Instruments). Droplet compositions including the applied ligand molecules (2 mM, 1 mM for ZOL) are denoted in Supplementary Table 2. Diffracting crystals grew within a few weeks and were cryoprotected by adding 1 µl of mother liquor containing 30% (v/v) glycerol prior to vitrification in liquid nitrogen.

Native datasets were collected at beamline X06SA (Swiss Light Source, Paul Scherrer Institute, Villigen, Switzerland) with synchrotron radiation (wavelength *λ* = 1.0 Å). Anomalous scattering was additionally recorded at 1.89 Å (Mn) or 0.92 Å (Br) for crystals grown in the presence of MnCl_2_ and 3-Br-GPP, respectively. Diffraction intensity data were evaluated by the XDS program and processed with XSCALE (Supplementary Table 3)^34^. Resolution limits were chosen to meet the following criteria: signal to noise ratio *I*/*σ*(*I*) > 2.0, statistic for the precision of the measurements of each unique reflection *R*_merge_ < 70% and redundancy of >3.0 (Supplementary Table 3). The initial structure of PcIDS1 was solved in space group *P*2_1_ (PDB 8A6U). The structure of FPPS from *Gallus gallus* (PDB 1UBX; ref. ^4^) was used for molecular replacement by Patterson search calculations with Phaser^35^. All other PcIDS1 structures were determined using the apo structure. Models were built, refined and completed with cofactors and ligands in COOT^36^ with intermittent, restrained refinements using REFMAC5 (ref. ^37^). Water molecules were positioned by ARP/wARP solvent^38^. TLS (Translation/Libration/Screw) and restrained refinements yielded adequate model parameters (Supplementary Table 3) as well as root-mean-square deviation (r.m.s.d.) values of bond lengths and angles (validated by PROCHECK^39^). To identify Mn and Br positions in the protein–ligand complex, anomalous difference Patterson maps were calculated with fast Fourier transformation^40^. The crystal structures were deposited in the RCSB Protein Data Bank (Supplementary Table 3).

### Figure illustration

To systematically characterize substrate coordination in PcIDS1, figures were prepared with Molscript/Bobscript^41^. Protein residues (grey, marked by a one-letter code) and ligands at the allylic (Al, cyan), homoallylic (HAl, purple) and allosteric (Allo, teal) sites are shown as sticks. Mg^2+^ ions (black), Mn^2+^ ions (pink) and water molecules (red) are depicted as balls with cluster interactions represented by black dashes. Structural superpositions with PyMol (ref. ^42^) highlight discrepancies at the active site (coloured and grey). Unless noted otherwise, all electron density maps are F_O_–F_C_ (green or blue mesh, contoured to 3.0 σ) or anomalous signal maps (pink for Mn and blue mesh for Br, 10.0 σ) with respective ligands omitted prior to phasing. The 2F_O_–F_C_ maps around Mg^2+^ ions are contoured to 1.0 σ and coloured grey. Calculations combine the observed diffraction data, F_O_, with the diffraction data calculated from the atomic model, F_C_; σ represents the density value above the average value.

### Annotations

The r.m.s.d. values were calculated for the Cα backbone with Top3D (ref. ^43^) and are annotated in Supplementary Table 4.

### Reporting summary

Further information on research design is available in the Nature Portfolio Reporting Summary linked to this article.

## Online content

Any methods, additional references, Nature Portfolio reporting summaries, source data, extended data, supplementary information, acknowledgements, peer review information; details of author contributions and competing interests; and statements of data and code availability are available at 10.1038/s41557-023-01235-9.

## Supplementary information

Supplementary Information Supplementary Figs. 1–11, Tables 1–4, references and additional source data.

Reporting Summary

Supplementary Data 1 Source data for Supplementary Fig. 1a.

Supplementary Data 2 Source data analysis for Supplementary Fig. 2.

Supplementary Data 3 Source data for Supplementary Fig. 2.

## Figures and Tables

**Extended Data Fig. 3:**
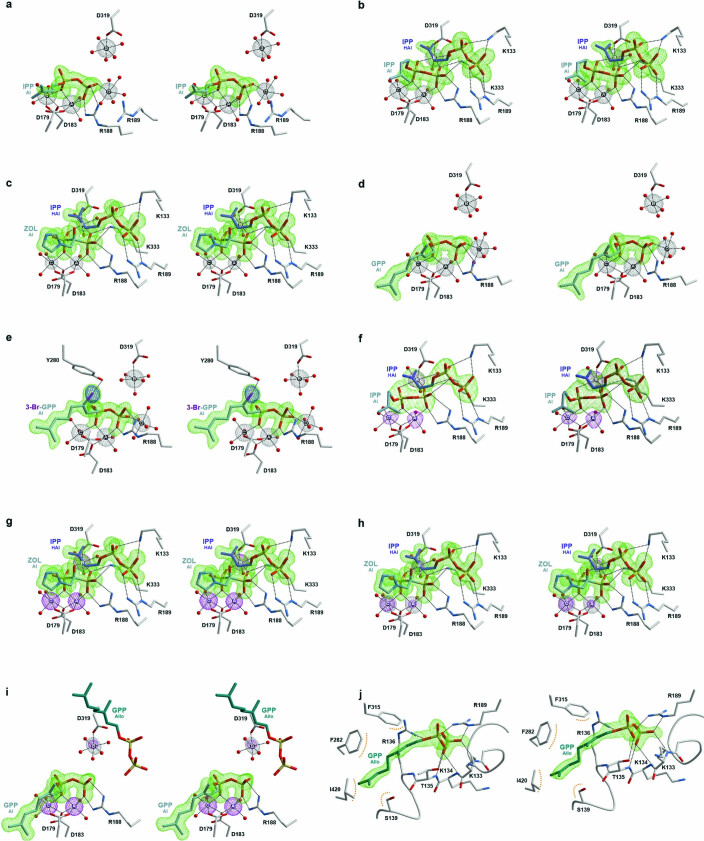
Stereo views of *Pc*IDS1 structures. **a-j**. All representations according to Fig. 3.

## Data Availability

Crystallographic data were deposited in the RCSB Protein Data Bank with the PDB identification numbers 8A6U, 8A6V, 8A6Z, 8A70, 8A73, 8A74, 8A78, 8A7A, 8A7B, 8A7C, 8A7J, 8A7K, 8A7L, 8A7R and 8A7U. All other data are available in the text or in the Supplementary Information.
