# Supplementary material for: Metal-dependent enzyme symmetry guides the biosynthetic flux of terpene precursors
Source: Nat Chem. 2023 Jun 12;15(8):1188–95. doi: 10.1038/s41557-023-01235-9 (PMC10396970; doi:10.1038/s41557-023-01235-9)

nature chemistry

Article https://doi.org/10.1038/s41557-023-01235-9

Metal-dependent enzyme symmetry guides the biosynthetic flux of terpene precursors

In the format provided by the authors and unedited

This PDF file includes:

Pages 1-14:
Supplementary Figure 1 to 11

Pages 15-22:
Supplementary Table 1 to 4

Page 23:
Supplementary References

Page 24:
Additional Source Data

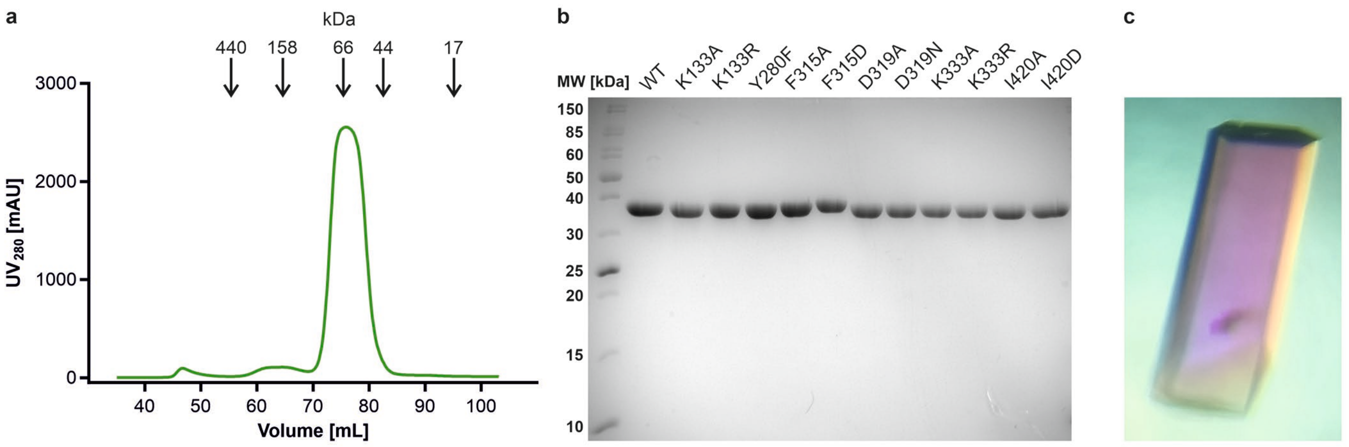

Supplementary Fig. 1. | Purification and crystallization of *Pc*IDS1. a, Size-exclusion chromatography of *Pc*IDS1 wild-type using a Superdex 200 16/60 column. The corresponding retention volumes of standard proteins are plotted according to the manufacturer's manual. *Pc*IDS1 forms a homodimer that elutes at 76 mL. **b**, Analysis by SDS-PAGE reveals that all purified protein variants closely match the expected size of 40 kDa. 5 µg of thawed protein were applied to each lane. Protein traces correspond to the results obtained directly after each purification. Small discrepancies are caused by irregularities in gel polymerisation. **c**, A typical protein crystal (200 x 50 x 50 µm) viewed under a light microscope with a polarisation filter.

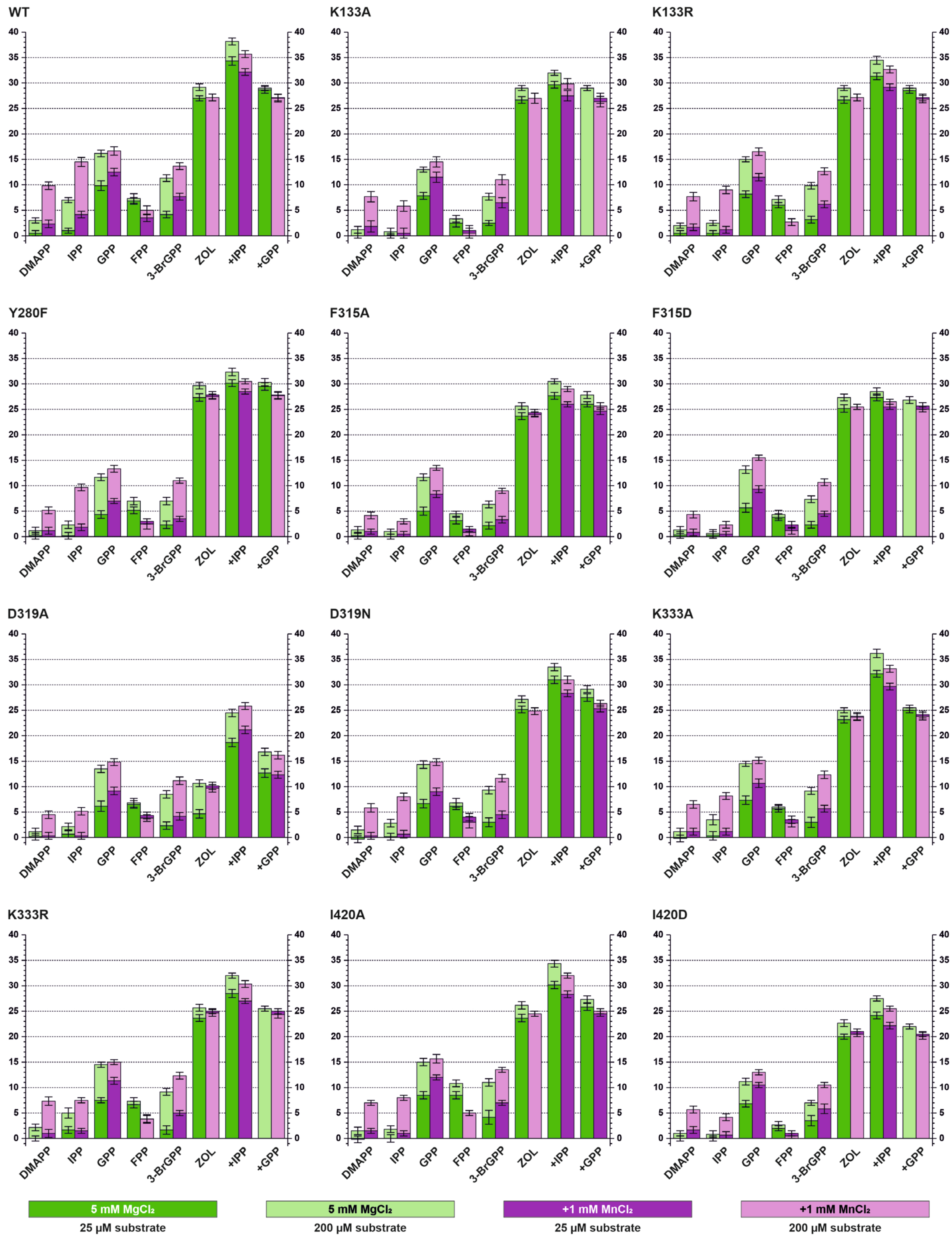

Supplementary Fig. 2. | Thermal stability of *Pc*IDS1 and protein variants in complex with different ligands. Bars represent the difference in mean melting temperature [°C] of *Pc*IDS1:ligand complexes compared to the respective apo enzyme. Ligands were applied at 25 µM (dark) and 200 µM (light green/purple). Error bars indicate the standard error of the mean for triplicate measurements.

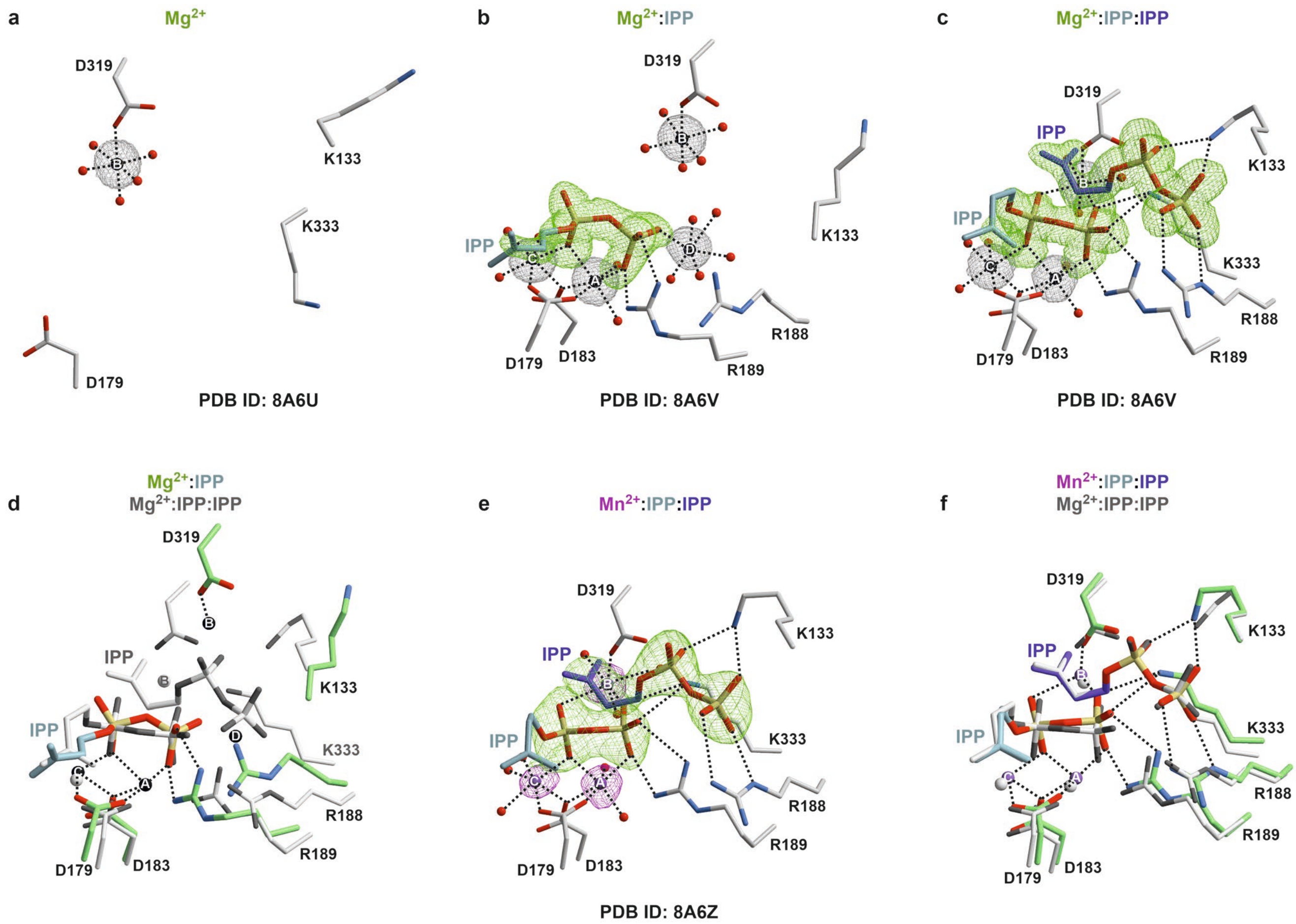

Supplementary Fig. 3. | *Pc*IDS1 in complex with IPP. a, *Pc*IDS1$_{apo}$: A pentahydrated Mg^{2+} in position B coordinates D319. The substrate loop between αD and αE is structurally distorted. **b,** *Pc*IDS1$_{Mg}$:IPP: The IPP$_{Al}$ ligand mimics the native substrate DMAPP with four Mg^{2+} (sites A-D) clustered around the allylic pyrophosphate (PP$_{Al}$) moiety (open state). The substrate loop is fully defined and R189 coordinates the β-phosphate ($P_β$). **c,** *Pc*IDS1$_{Mg}$:IPP:IPP: The IPP$_{HAl}$ substrate is coordinated by K133, R136 and R189, and forces the allylic ligand into an eclipsed conformation (closed state). The SARM moves Mg^{2+}_B towards PP$_{Al}$ and interacts with both phosphates. Mg^{2+}_D is displaced from $P_β$ that now contacts K333. **d,** Superposition of *Pc*IDS1$_{Mg}$:IPP and *Pc*IDS1$_{Mg}$:IPP:IPP: Binding of IPP$_{HAl}$ induces major rearrangements of the SARM. The coordination spheres of Mg^{2+}_A and Mg^{2+}_C as well as the phosphor atoms of PP$_{Al}$ remain at their original positions. **e,** *Pc*IDS1$_{Mn}$:IPP:IPP: In presence of Mn^{2+}, sites A-C are occupied by the heavy metal ions (HMI). In contrast to *Pc*IDS1$_{Mg}$:IPP:IPP, both subunits of the crystal structure are in the closed state. **f,** Superposition of *Pc*IDS1$_{Mn}$:IPP:IPP and *Pc*IDS1$_{Mg}$:IPP:IPP: No significant discrepancies are observed. Colour coding is described in *Materials and Methods*.

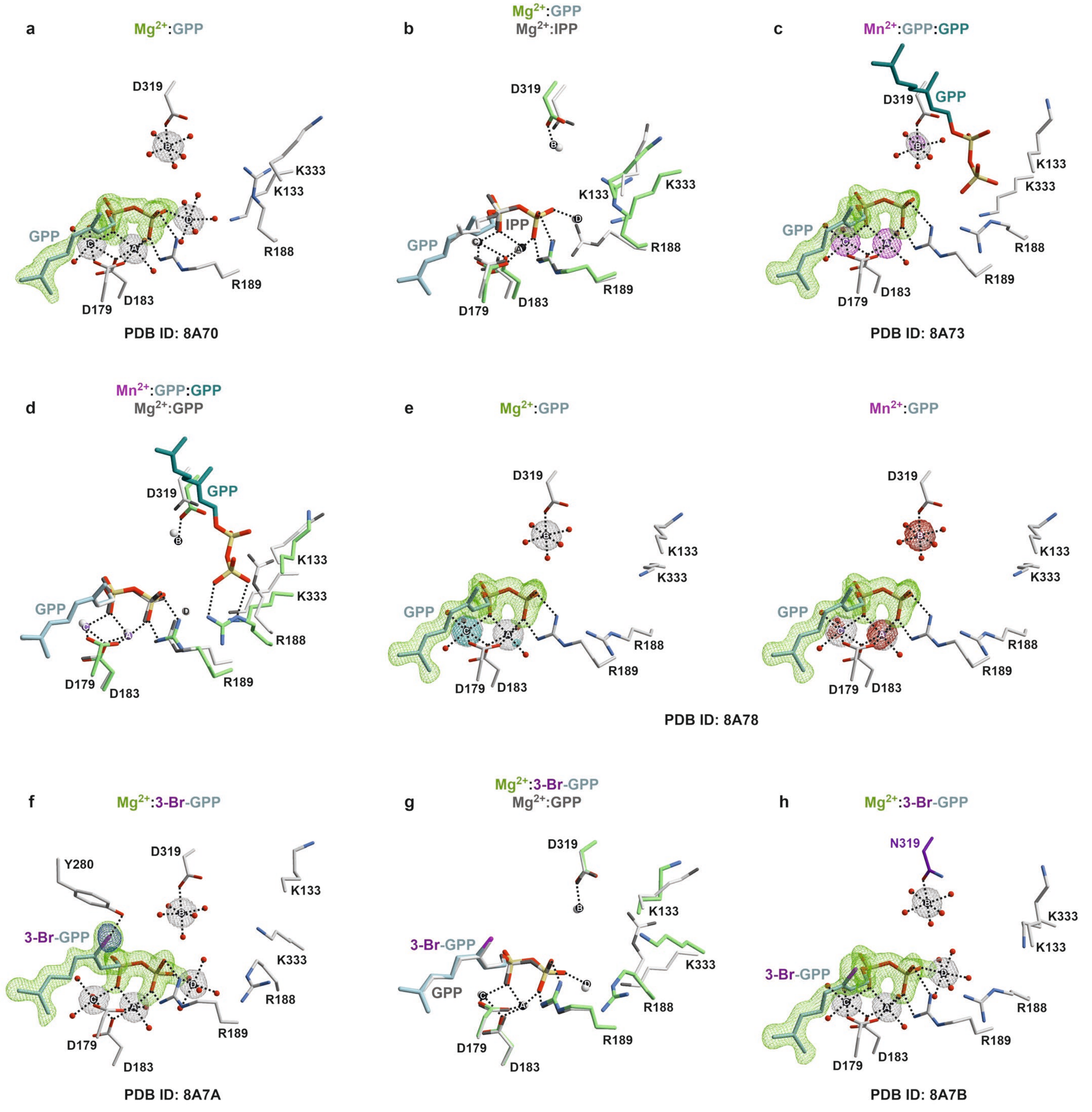

Supplementary Fig. 4. | *Pc*IDS1 in complex with GPP and 3-Br-GPP. a, *Pc*IDS1$_{Mg}$:GPP: Four Mg^{2+} ions cluster around GPP$_{Al}$ that adapts a similar orientation to IPP$_{Al}$. **b,** Superposition of *Pc*IDS1$_{Mg}$:GPP and *Pc*IDS1$_{Mg}$:IPP: No significant discrepancies are observed between the allylic pockets. In absence of IPP$_{HAl}$, K133, R188 and K333 display high temperature factors. **c,** *Pc*IDS1$_{Mn}$:GPP:GPP: Mn^{2+}occupies sites A and C with low occupancy at site B where Mg^{2+} remains the dominating metal ion. Notably, the pentahydrated $Mg^{2+}{}_{D}$ is absent. R188 provides an interface for GPP$_{Allo}$ binding. **d,** Superposition of *Pc*IDS1$_{Mn}$:GPP:GPP and *Pc*IDS1$_{Mg}$:GPP: In absence of $Mg^{2+}{}_{D}$, the SARM shifts towards PP$_{Al}$ by 0.7 Å. **e,** *Pc*IDS1_F315A$_{Mg}$:GPP: Crystals were obtained in presence of Mn^{2+}; $Mg^{2+}{}_{D}$ is not observed.

Left panel: If metal positions A-C are assigned as Mg^{2+}, positive F_O-F_C electron density (cyan) emerges at site C. Right panel: If Mn^{2+} is placed at A-C, negative F_O-F_C signals (red) are observed at A and B. Therefore, A and C are assigned as Mg^{2+} and C as Mn^{2+}. **f,** *Pc*IDS1$_{Mg}$:3-Br-GPP: The inhibitor 3-Br-GPP binds to the Al site and is surrounded by four Mg^{2+} ions. The anomalous bromine electron density is shown in blue. **g,** Superposition of *Pc*IDS1$_{Mg}$:3-Br-GPP and *Pc*IDS1$_{Mg}$:GPP: The binding mode of the inhibitor matches GPP$_{Al}$. **h,** *Pc*IDS1_D319N$_{Mg}$:3-Br-GPP: The pentahydrated $Mg^{2+}{}_B$ remains coordinated if D319 is exchanged to asparagine. Colour coding is described in *Materials and Methods*.

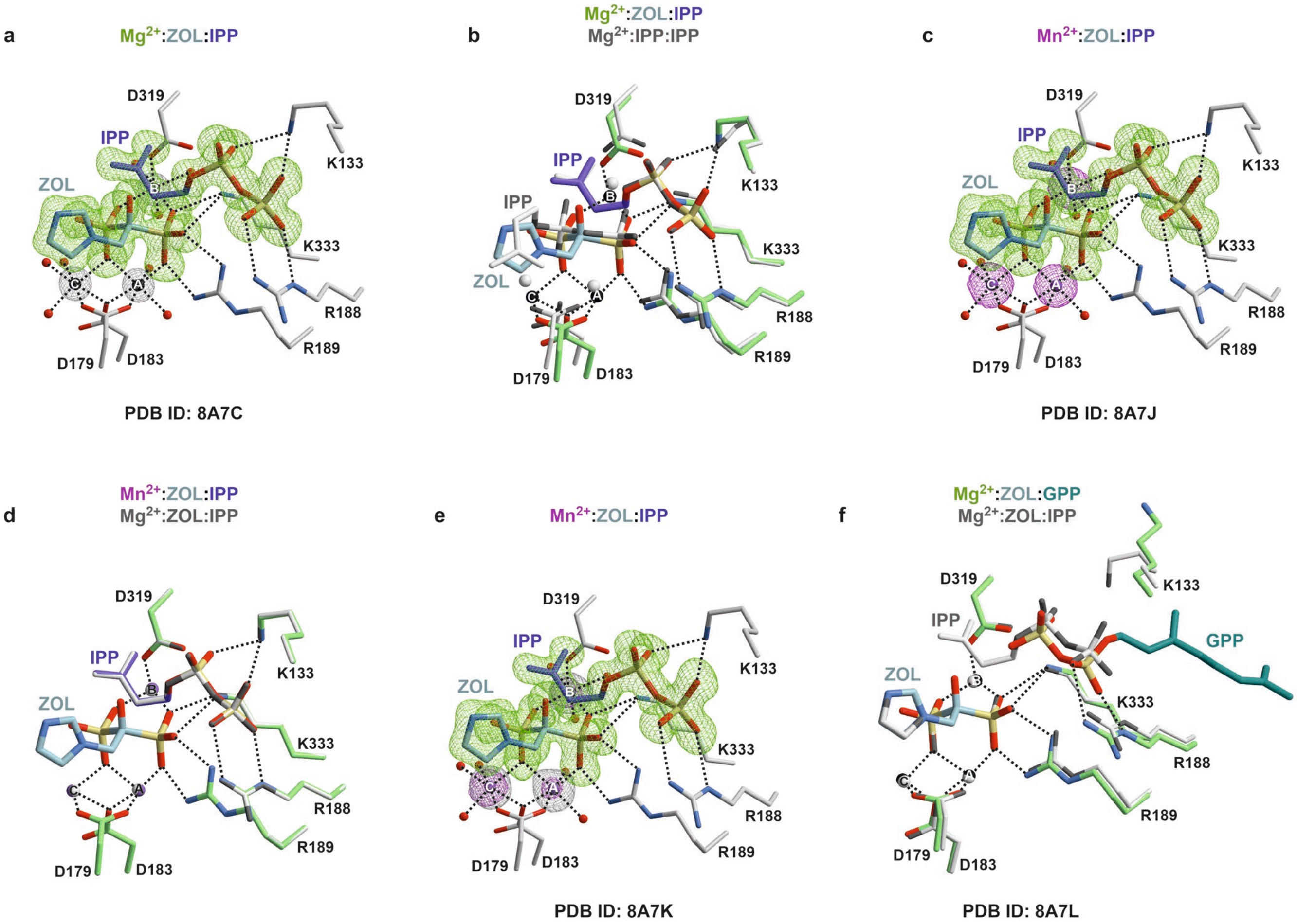

Supplementary Fig. 5. | *Pc*IDS1 in complex with zoledronic acid (ZOL). a, *Pc*IDS1$_{\mathrm{Mg}}$:ZOL:IPP: The bisphosphonate (BP) of ZOL mimics the PP$_{\mathrm{Al}}$ moiety and clusters three Mg^{2+} ions in presence of IPP$_{\mathrm{HAl}}$. **b,** Superposition of *Pc*IDS1$_{\mathrm{Mg}}$:ZOL:IPP and *Pc*IDS1$_{\mathrm{Mg}}$:IPP:IPP: The inhibitor induces a uniform arrangement of the active site. The eclipsed orientation of BP causes minor rearrangements of the metal positions and residues of the active site. **c,** *Pc*IDS1$_{\mathrm{Mn}}$:ZOL:IPP: In presence of Mn^{2+}, sites A-C are occupied by the HMIs at full occupancy. **d,** Superposition of *Pc*IDS1$_{\mathrm{Mn}}$:ZOL:IPP and *Pc*IDS1$_{\mathrm{Mg}}$:ZOL:IPP: No significant discrepancies are observed. **e,** *Pc*IDS1$_{\mathrm{Mn/Mg}}$:ZOL:IPP: In a 50:1 stoichiometry, Mg^{2+} displaces Mn^{2+} from sites A and B, but to lesser extent at position C. **f,** Superposition of *Pc*IDS1$_{\mathrm{Mg}}$:ZOL:GPP and *Pc*IDS1$_{\mathrm{Mg}}$:ZOL:IPP: The strong inhibitor ZOL prevents binding of GPP$_{\mathrm{Al}}$. GPP binds to HAl with inverted α- and β-positions compared to IPP$_{\mathrm{HAl}}$. The C_{10} prenyl unit of GPP remains surface exposed. Colour coding is described in *Materials and Methods*.

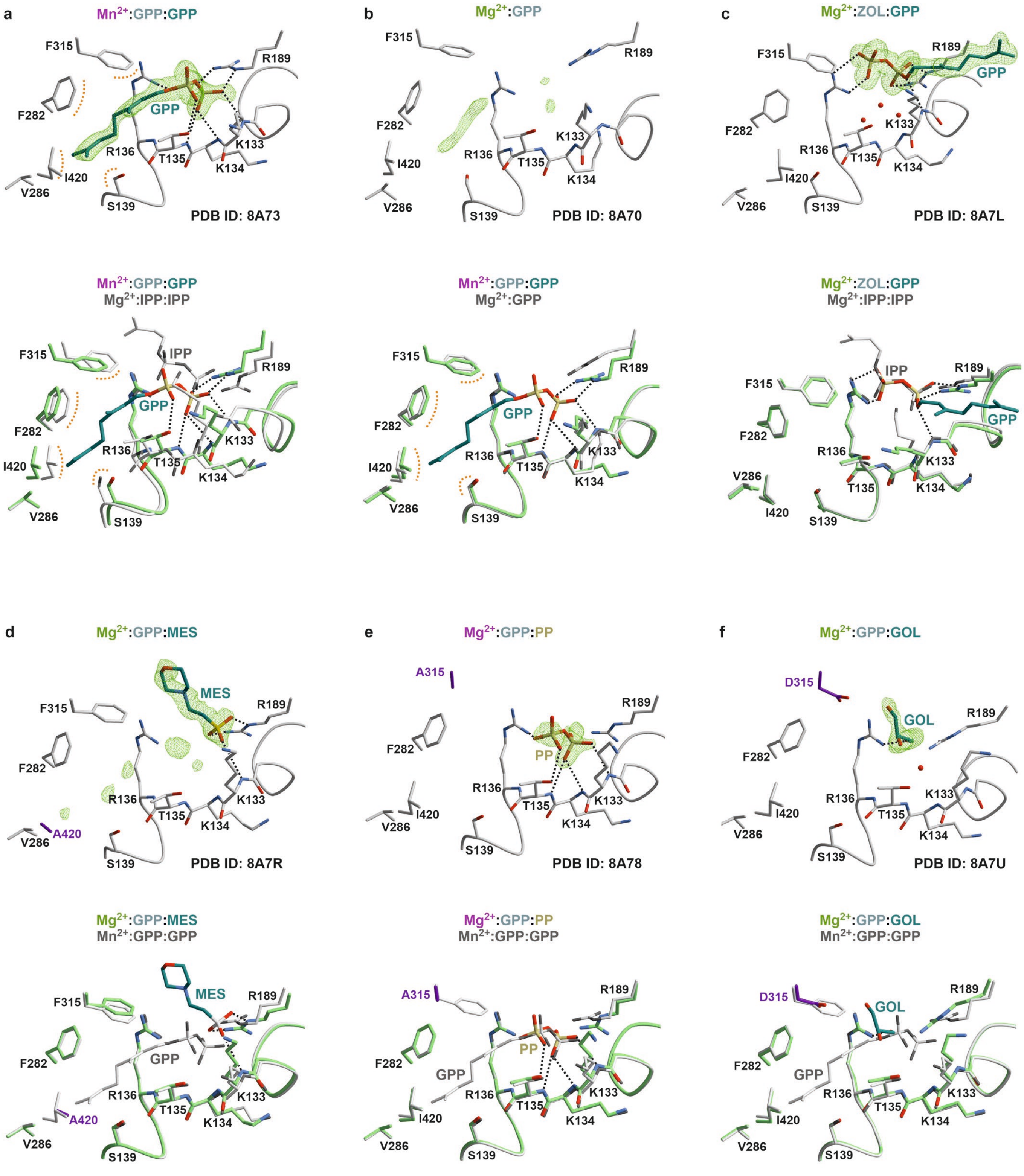

Supplementary Fig. 6. | Structural analysis of the allosteric site in *Pc*IDS1. a, *Pc*IDS1$_{Mn}$:GPP:GPP (top): In presence of Mn^{2+}, GPP binds to an allosteric pocket between αC, αG and αJ. Hydrophobic and steric interactions are indicated by dashed orange lines. Superposition of *Pc*IDS1$_{Mn}$:GPP:GPP and *Pc*IDS1$_{Mg}$:IPP:IPP (bottom): The HAl

site is rearranged to accommodate GPP_{Allo} that acts as a competitive inhibitor for IPP_{HAl}. The PP_{Allo} moiety coordinates R189 and anchors its P_{β} to the N-terminus of αC through backbone amide interactions. Additional π-π-stacking between the prenyl double bonds and F282 as well as F315 mediates ligand selectivity. **b,** *Pc*IDS1$_{Mg}$:GPP (top): GPP_{Allo} is absent if the metal sites at GPP_{Al} are occupied by Mg^{2+}. The elongated, diffuse signal in the allosteric channel indicates unspecific affinity for apolar molecules. Superposition of *Pc*IDS1$_{Mn}$:GPP:GPP and *Pc*IDS1$_{Mg}$:GPP (bottom): Basic residues involved in PP_{HAl} interactions remain flexible in the Mg^{2+} structure. **c,** *Pc*IDS1$_{Mg}$:ZOL:GPP (top): ZOL_{Al} locks the subunit in the closed state and therefore recruits GPP to HAl in a non-physiological, allosteric coordination. Superposition of *Pc*IDS1$_{Mg}$:ZOL:GPP and *Pc*IDS1$_{Mg}$:IPP:IPP (bottom): The PP_{HAl} moieties in both ligands display a uniform binding mode with inverted α- and β-positions. **d,** *Pc*IDS1_I420A$_{Mg}$:GPP:MES (top): The allosteric channel of this variant is enlarged and no longer binds GPP_{Allo}. A 2-(N-morpholino)ethanesulfonic acid (MES) molecule occupies the HAl site and prevents binding of Mg^{2+}_{D}. Superposition of *Pc*IDS1_I420A$_{Mg}$:GPP:MES and *Pc*IDS1$_{Mn}$:GPP:GPP (bottom): In absence of GPP_{Allo}, F315 rotates to its native position and R189 faces the sulfone moiety of MES. **e,** *Pc*IDS1_F315A$_{Mg}$:GPP:PP (top): Diffuse electron density indicates the presence of PP_{Allo}. The GPP prenyl moiety is distorted in absence of the F315 phenyl group. Superposition of *Pc*IDS1_F315A$_{Mg}$:GPP:PP and *Pc*IDS1$_{Mn}$:GPP:GPP (bottom): F315 conveys specificity towards C_{10} prenyl units, whereas the N-terminus of αC recruits PP groups. **f,** *Pc*IDS1_F315D$_{Mg}$:GPP:GOL (top): Binding of GPP_{Allo} is prohibited by D315 and a glycerol (GOL) molecule occupies the HAl site. Superposition of *Pc*IDS1_F315D$_{Mg}$:GPP:GOL and *Pc*IDS1$_{Mn}$:GPP:GPP (bottom): The integrity of the HAl and Allo sites remains conserved in F315D. Note, crystals structures of *Pc*IDS1 variants in **d-f** were obtained in presence of Mn^{2+}, but GPP_{Al} is coordinated by Mg^{2+}. Colour coding is described in *Materials and Methods*.

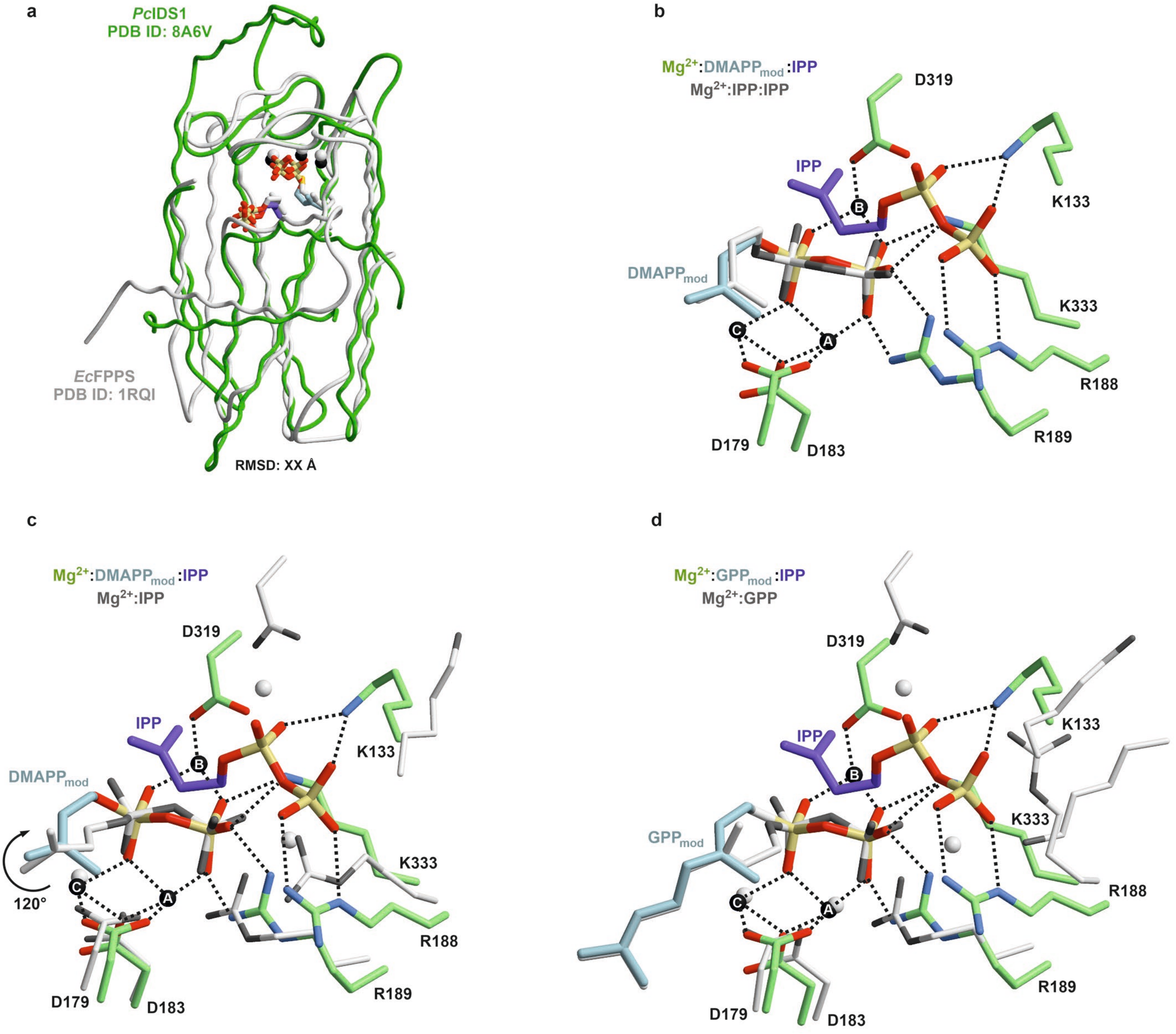

Supplementary Fig. 7. | Modelling of $DMAPP_{Al}$ and GPP_{Al} in the closed state. a, The *Pc*$IDS1_{Mg}$:IPP:IPP subunit (green) displays structural conservation to the *E. coli* FPPS (grey) complex with dimethylallyl S-thiolodiphosphate (DMSPP) and IPP (*1*). **b**, The *Pc*IDS1 Al site with $DMAPP_{mod}$ (modelled from DMSPP) and IPP_{HAl} illustrates the binding mode of the two native substrates. $DMAPP_{mod}$ adapts the same conformation as IPP_{Al} in the closed state. **c,** The superposition of *Pc*$IDS1_{Mg}$:$DMAPP_{mod}$:IPP and *Pc*$IDS1_{Mg}$:IPP highlights a 120° clockwise rotation of the $DMAPP_{mod}$ prenyl moiety in presence of IPP_{HAl}. **d,** The superposition of *Pc*$IDS1_{Mg}$:GPP_{mod}:IPP and *Pc*$IDS1_{Mg}$:GPP shows that GPP undergoes the same reorientation as $DMAPP_{Al}$ during the reaction. GPP_{Al} was modelled based on $DMAPP_{mod}$ and *Pc*$IDS1_{Mg}$:GPP. Colour coding is described in *Materials and Methods*.

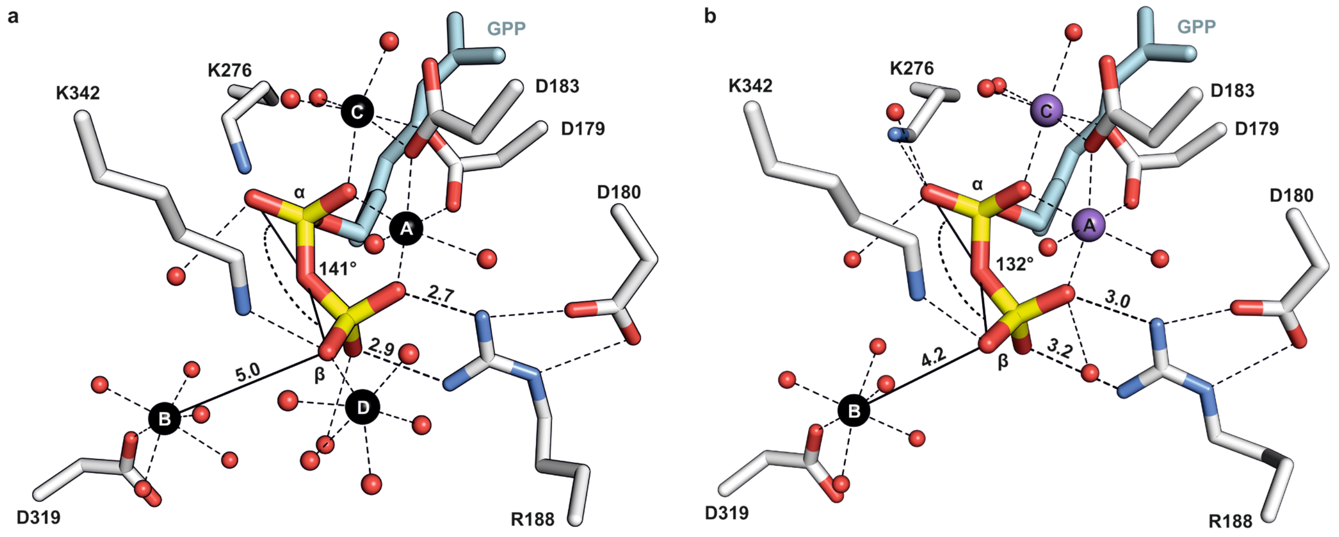

Supplementary Fig. 8. | Differences in coordination of GPP_{Al} in presence of Mg^{2+} and Mn^{2+}. a, *Pc*$IDS1_{Mg}$:GPP displays four metal-binding sites around PP_{Al}. P_{β} forms strong H-bonds with R188 and coordinates the pentahydrated $Mg^{2+}{}_{D}$. **b,** *Pc*$IDS1_{Mn}$:GPP:GPP: The Lewis acidity of HMIs at sites A and C decreases the charge density of P_{β} and weakens the interaction with R188. Notably, $Mg^{2+}{}_{D}$ is absent and the distance between PP_{Al} and $Mg^{2+}{}_{B}$ decreases by 0.8 Å. These rearrangements promote transition into the eclipsed conformation of the allylic ligand upon IPP_{HAl} binding. Colour coding is described in *Materials and Methods*.

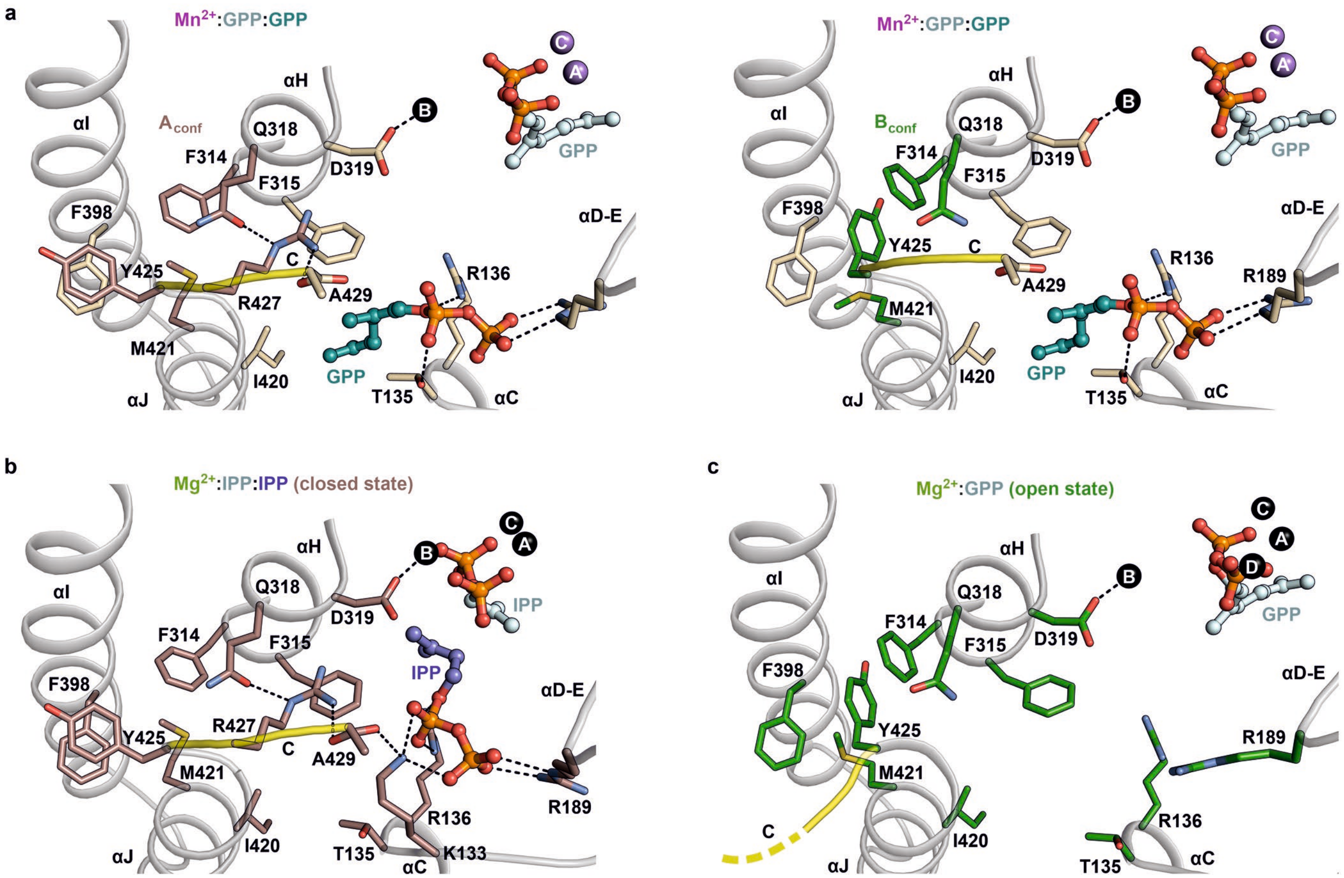

Supplementary Fig. 9. | Architecture of the allosteric GPP-binding site. The *Pc*IDS1$_{Mn}$:GPP structure binds a second GPP molecule in an allosteric binding pocket formed by αC, αG and αJ. The C-terminus (yellow) turns towards the active site and shows alternative conformations for F314, Q318, M421, Y425 and R427 (static residues are coloured in light brown). **a**, *Pc*IDS1$_{Mn}$:GPP:GPP: Left: Residues in conformation A correspond to the closed state and are shown in dark brown (A$_{conf}$). F315 forms cation-π interactions with R427 that coordinates Q318 and positions the C-terminus. The loop region is further stabilised by π-stacking between Y425 and F398. Right: Residues in B$_{conf}$ adapt orientations of the open state (dark green). Therefore, GPP$_{Allo}$ arrests the C-terminus between the closed (**b,** *Pc*IDS1$_{Mg}$:IPP:IPP) and the open state (**c,** *Pc*IDS1$_{Mg}$:GPP). Colour coding is described in *Materials and Methods*.

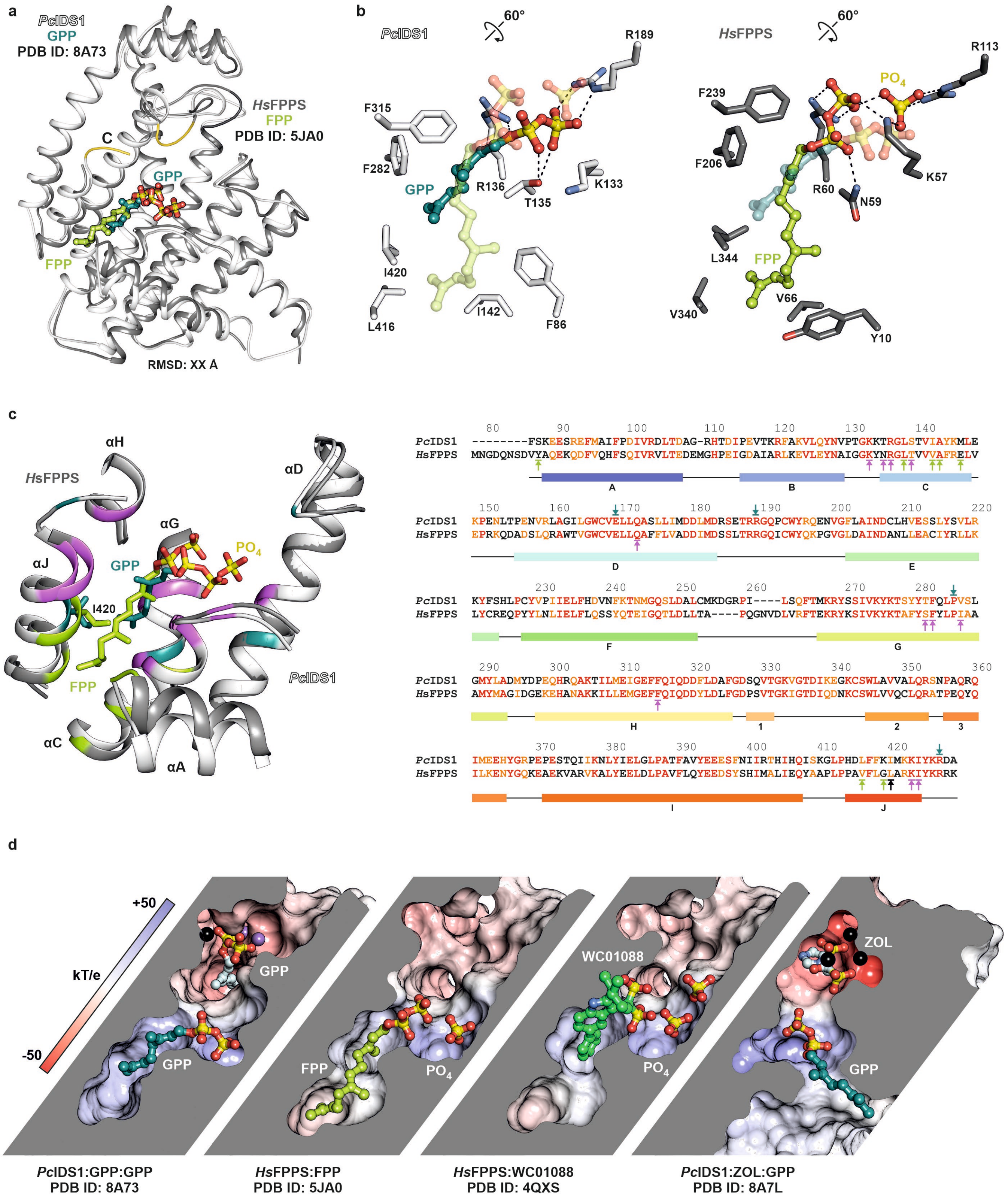

Supplementary Fig. 10. | Allosteric binding in *Pc*IDS1 compared to *Hs*FPPS. a, Superposition of *Pc*IDS1$_{Mn}$:GPP:GPP (white) and FPPS:FPP$_{Allo}$ from *Homo sapiens* (*Hs*FPPS, dark grey, (*2*)): The protein backbones

are drawn as cartoon, GPP (teal) and FPP (green) are shown as balls-and-sticks. Structural features introduced by GPP_{Allo} binding to *Pc*IDS1 are highlighted in yellow. **b,** Zoom of the allosteric site in *Pc*IDS1 (left) and *Hs*FPPS (right). While GPP_{Allo} contacts two arginine residues (R136 and R189) of the HAl site, the equivalent residues (R60 and R113) in FPP_{Allo} interact with P_{β} and an additional phosphate ion. Consequently, the prenyl tail of FPP is flipped compared to GPP and orients the 3-methyl group towards the protein core. I420 in *Pc*IDS1 forms the bottom of the hydrophobic GPP channel, while access to a more spacious specificity pocket for FPP_{Allo} is provided by L344 in *Hs*FPPS. **c,** Left: Detail view of the Allo site of **a** with residues (< 5 Å distance to the respective ligand) highlighted in teal (GPP-specific), green (FPP-specific) and pink (unspecific). Right: The sequence alignment displays significant relation between both enzymes. The degree of conservation is shown in orange (conservative substitution) and red (conserved). Numbering and secondary structure elements (helices A to J) correspond to *Pc*IDS1. Arrows indicate the sites of allosteric interactions according to the colour scheme on the left and I420/L344 are highlighted in black. **d,** Cross sections of the *Pc*IDS1 and *Hs*FPPS active sites in complex with different allosteric ligands. The electrostatic surfaces (-50 to 50 kT/e) show the acidic character of the Al, as well as the basic nature of the HAl sites. While the allosteric channels of both proteins are similar in size and length, small changes in the hydrophobic network convey ligand specificity. The substance WC01088 in *Hs*FPPS (*3*) occupies the same branch of the cavity as GPP_{Allo} and indicates that inhibitors can selectively target the Allo site. Strong inhibitors such as ZOL_{Al} enforce the closed state of the subunit (right panel) and prohibit access to the Allo site. GPP can still bind to the HAl site with the PP moiety by pushing its C_{10} prenyl unit towards the protein surface. Colour coding is described in *Materials and Methods*.

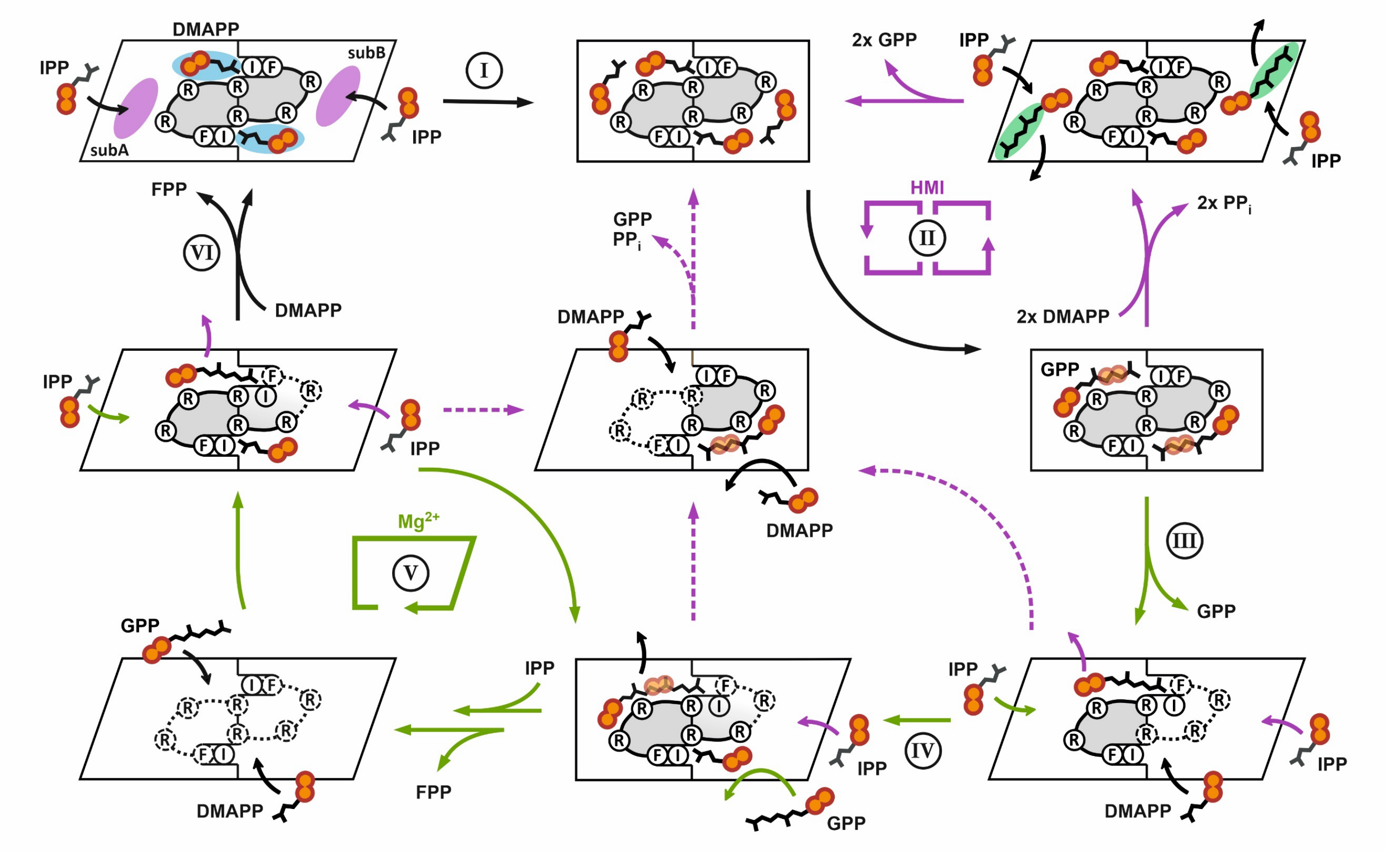

Supplementary Fig. 11 | Reaction network of *Pc*IDS1. The two subunits of the homodimer (sub_A and sub_B) are depicted in either the open (rhombic) or closed state (rectangle), respectively. Substrate loops and crucial residues are shown at the dimer interface in their defined (grey) or unstructured conformation (white). Substrate coordination is highlighted by arrows in black (indifferent to metal type), green (Mg^{2+}) and pink (HMI). The intersubunit cooperativity recruits DMAPP to Al (cyan) followed by IPP to HAl (purple) in both subunits (**I**). The elongation proceeds to GPP which is the preferred product of HMIs that increase IPP_{HAl} affinity and accelerate catalysis (**II**). At high concentration, GPP binds to the allosteric site (teal) and slows down catalysis. Mg^{2+} stabilises the open state with GPP_{Al} bound to one subunit, while the adjacent substrate loop retains affinity for $DMAPP_{Al}$ (**III**). FPP emerges in the asymmetric complex upon IPP_{HAl} binding (**IV**). The final product is released and Mg^{2+} drives FPP formation in the single-subunit catalyst (**V**) until GPP levels decrease (**VI**). An influx of HMIs counteracts GPP_{Al} binding by recruiting IPP_{HAl} to the adjacent subunit (dashed pink arrows).

Supplementary Table 1. Primer sequences for *Pc*IDS1 mutagenesis with altered codons highlighted in red

Mutant	Forward primer (5' → 3')	Reverse primer (5' → 3')
K133A	TAC AAC GTT CCG ACC GGT GCA AAA ACC CGT GGT CTG TC	GA CAG ACC ACG GGT TTT TGC ACC GGT CGG AAC GTT GTA
K133R	AAC GTT CCG ACC GGT AGA AAA ACC CGT GGT CTG	CAG ACC ACG GGT TTT TCT ACC GGT CGG AAC GTT
Y280F	A TAC AAA ACC TCT TAC TTC ACC TTC CAG CTG CCG G	C CGG CAG CTG GAA GGT GAA GTA AGA GGT TTT GTA T
F315A	G ATG GAA ATC GGT GAA TTC GCC CAG ATC CAG GAC GAC TTC	GAA GTC GTC CTG GAT CTG GGC GAA TTC ACC GAT TTC CAT C
F315D	G ATG GAA ATC GGT GAA TTC GAC CAG ATC CAG GAC GAC TTC	GAA GTC GTC CTG GAT CTG GTC GAA TTC ACC GAT TTC CAT C
D319A	C TTC CAG ATC CAG GCC GAC TTC CTG GAC G	C GTC CAG GAA GTC GGC CTG GAT CTG GAA G
D319N	A TTC TTC CAG ATC CAG AAC GAC TTC CTG GAC GC	GC GTC CAG GAA GTC GTT CTG GAT CTG GAA GAA T
K333A	GT GAC TCT CAG GTT ACC GGT GCA GTT GGT ACC GAC ATC A	T GAT GTC GGT ACC AAC TGC ACC GGT AAC CTG AGA GTC AC
K333R	AC TCT CAG GTT ACC GGT AGA GTT GGT ACC GAC AT	AT GTC GGT ACC AAC TCT ACC GGT AAC CTG AGA GT
I420A	G CCG CAC GAC CTG TTC TTC AAA GCC ATG AAG AAG ATC TAC	GTA GAT CTT CTT CAT GGC TTT GAA GAA CAG GTC GTG CGG C
I420D	G CCG CAC GAC CTG TTC TTC AAA GAC ATG AAG AAG ATC TAC	GTA GAT CTT CTT CAT GTC TTT GAA GAA CAG GTC GTG CGG C

Supplementary Table 2. Sitting-drop crystallization parameters of reported datasets.

Protein	Conc. [mg/mL]	Protein-reservoir ratio [µL/µL]	Reservoir	Cofactors Ligands
*Pc*IDS1$_{apo}$	20	0.2 + 0.2	0.1 M Tris, pH 8.5<br>0.2 M $MgCl_2$<br>20% PEG 8000	$MgCl_2$
*Pc*IDS1$_{Mg}$<br>IPP:IPP	20	0.2 + 0.2	0.1 M Bis-Tris, pH 6.5<br>0.2 M $MgCl_2$<br>25% PEG 3350	$MgCl_2$<br>IPP
*Pc*IDS1$_{Mn}$<br>IPP:IPP	20	0.2+0.1	0.1 M Bis-Tris, pH 6.5<br>0.2 M $MgCl_2$<br>26% PEG 3350	$MgCl_2$/$MnCl_2$<br>IPP
*Pc*IDS1$_{Mg}$<br>GPP	20	0.3+0.1	0.1 M MES, pH 6.5<br>0.15 M $MgCl_2$<br>28% PEG 4000	$MgCl_2$<br>GPP
*Pc*IDS1$_{Mn}$<br>GPP:GPP	20	0.2+0.2	0.4 M Potassium formate<br>19% PEG 3350	$MgCl_2$/$MnCl_2$<br>GPP
*Pc*IDS1_F315A$_{Mg}$<br>2GPP	20	0.3+0.1	0.1 M MES, pH 6.5<br>0.2 M $MgCl_2$<br>25% PEG 4000	$MgCl_2$<br>GPP
*Pc*IDS1_F315A$_{Mg/Mn}$<br>GPP:PP	20	0.2+0.2	0.1 M MES, pH 6.5<br>0.6 M NaCl<br>20% PEG 4000	$MgCl_2$/$MnCl_2$<br>GPP
*Pc*IDS1$_{Mg}$<br>3-Br-GPP	20	0.2+0.2	0.1 M MES, pH 6.5<br>0.2 M $MgCl_2$<br>25% PEG 4000	$MgCl_2$<br>3-Br-GPP
*Pc*IDS1_D319N$_{Mg}$<br>3-Br-GPP	20	0.2+0.2	0.1 M MES, pH 6.5<br>0.2 M $MgCl_2$<br>25% PEG 4000	$MgCl_2$<br>3-Br-GPP
*Pc*IDS1$_{Mg}$<br>ZOL:IPP	20	0.2 + 0.2	0.1 M Bis-Tris, pH 5.5<br>0.2 M NaCl<br>19% PEG 3350	$MgCl_2$<br>ZOL<br>IPP
*Pc*IDS1$_{Mn}$<br>ZOL:IPP	20	0.2 + 0.2	0.1 M Bis-Tris, pH 5.5<br>0.1 M NaCl<br>23% PEG 3350	$MgCl_2$/$MnCl_2$<br>ZOL<br>IPP
*Pc*IDS1$_{Mg/Mn}$<br>ZOL:IPP	20	0.2 + 0.1	0.1 M Bis-Tris, pH 6.5<br>0.1 M $MgCl_2$<br>26% PEG 3350	$MgCl_2$ >> MnCl2<br>ZOL<br>IPP
*Pc*IDS1$_{Mg}$<br>ZOL:GPP	20	0.2+0.2	0.1 M Bis-Tris, pH 6.5<br>45% PEG 400	$MgCl_2$<br>ZOL<br>GPP
*Pc*IDS1_I420A$_{Mg}$<br>GPP:MES	20	0.2+0.1	0.1 M MES, pH 6.5<br>0.2 M $CaCl_2$<br>30% PEG 4000	$MgCl_2$/$MnCl_2$<br>GPP
*Pc*IDS1_F315D$_{Mg}$<br>GPP:GOL	20	0.2+0.2	0.1 M Tris, pH 8.5<br>0.5 M LiCl<br>28% PEG 6000	$MgCl_2$<br>GPP

Supplementary Table 3. X-ray data collection and refinement statistics

	*Pc*IDS1$_{apo}$	*Pc*IDS1$_{Mg}$ IPP:IPP	*Pc*IDS1$_{Mn}$ IPP:IPP	*Pc*IDS1$_{Mn}$ IPP:IPP ano
**Crystal parameters**				
Space group	$P2_1$	$P2_1$	$P2_12_12_1$	$P2_12_12_1$
Cell constants	a=59.2 Å<br>b=78.3 Å<br>c=89.6 Å<br>β=101.7°	a=57.4 Å<br>b=72.4 Å<br>c=84.9 Å<br>β=101.5°	a=58.0 Å<br>b=187.0<br>c=213.3 Å	a=58.0 Å<br>b=187.0<br>c=213.3 Å
Subunits / AU[a]	2	2	6	6
**Data collection**				
Beam line	X06SA, SLS	X06SA, SLS	X06SA, SLS	X06SA, SLS
Wavelength (Å)	1.0	1.0	1.0	1.89
Resolution range (Å)[b]	30-1.65 (1.75-1.65)	30-1.9 (2.0-1.9)	30-2.4 (2.5-2.4)	30-2.5 (2.6-2.5)
No. observations	292081	164221	606763	575468
No. unique reflections[c]	94381	52944	90473	148792[*]
Completeness (%)[b]	98.0/98.4	98.3/98.2	98.7/99.0	96.1/93.3
R_{merge} (%)[b, d]	3.5/48.8	5.3/59.3	13.7/59.6	12.7/58.6
I/σ (I)[b]	16.4/2.4	11.8/2.0	10.5/3.8	7.3/2.0
**Refinement (REFMAC5)**				
Resolution range (Å)	15-1.65	15-1.9	15-2.4	
No. refl. working set	89539	50190	85538	
No. refl. test set	4712	2642	4502	
No. non hydrogen	5786	5690	17387	
No. of Mg^{2+}/Mn^{2+}	2/0	7/0	0/18	
No. of ligand atoms	0	42	168	
Solvent	594	180	446	
R_{work}/R_{free} (%)[e]	15.3/19.4	16.4/20.2	22.4/26.8	
r.m.s.d. bond (Å) / (angle)[f]	0.005/1.2	0.002/1.2	0.003/1.2	
B-factor (Å^2)				
- Protein	38.5	35.7	36.5	
- Ligand/Ion	37.6	34.2	32.1	
- Solvent	54.4	38.4	45.2	
Ramachandran Plot (%)[g]	97.8/2.2/0	98.8/1.2/0	97.4/2.6/0	
PDB accession code	**8A6U**	**8A6V**	**8A6Z**	

[a] Asymmetric unit
[b] The values in parentheses for resolution range, completeness, R_{merge} and I/σ (I) correspond to the highest resolution shell
[c] Data reduction was carried out with XDS and from a single crystal. Friedel pairs were treated as identical (or individual[*]) reflections
[d] $R_{merge}(I) = \Sigma_{hkl}\Sigma_j \mid I(hkl)_j - \langle I(hkl)\rangle \mid / \Sigma_{hkl} \Sigma_j I(hkl)_j$, where $I(hkl)_j$ is the jth measurement of the intensity of reflection hkl and <I(hkl)> is the average intensity
[e] $R = \Sigma_{hkl} \mid |F_{obs}| - |F_{calc}| \mid / \Sigma_{hkl} |Fobs|$, where R_{free} is calculated without a sigma cut off for a randomly chosen 5% of reflections, which were not used for structure refinement, and R_{work} is calculated for the remaining reflections
[f] Deviations from ideal bond lengths/angles
[g] Percentage of residues in favoured region / allowed region / outlier region

	***Pc*IDS1$_{Mg}$ GPP**	***Pc*IDS1$_{Mn}$ GPP**	***Pc*IDS1$_{Mn}$ GPP ano**	***Pc*IDS1_F315A$_{Mg}$ 2GPP**
**Crystal parameters**				
Space group	P2$_1$	P2$_1$	P2$_1$	P2$_1$
Cell constants	a=59.3 Å	a=59.1 Å	a=59.1 Å	a=59.2 Å
	b=77.3 Å	b=78.4 Å	b=78.4 Å	b=78.3 Å
	c=88.7 Å	c=87.3 Å	c=87.3 Å	c=89.3 Å
	β=101.4°	β=101.7°	β=101.7°	β=101.8°
Subunits / AU[a]	2	2	2	2
**Data collection**				
Beam line	X06SA, SLS	X06SA, SLS	X06SA, SLS	X06SA, SLS
Wavelength (Å)	1.0	1.0	1.89	1.0
Resolution range (Å)[b]	30-2.1	30-1.6	30-2.15	30-1.9
	(2.2-2.1)	(1.7-1.6)	(2.25-2.15)	(2.0-1.9)
No. observations	127936	271562	265577	209553
No. unique reflections[c]	44714	97381	81096[*]	61843
Completeness (%)[b]	97.2/96.7	94.7/97.8	97.1/91.9	98.3/99.4
R_{merge} (%)[b, d]	7.8/56.2	5.2/52.2	6.6/36.2	9.4/53.8
I/σ (I)[b]	8.1/2.0	10.1/2.0	10.8/2.7	6.9/2.4
**Refinement (REFMAC5)**				
Resolution range (Å)	15-2.1	15-1.6		15-1.9
No. refl. working set	42358	92404		58621
No. refl. test set	2229	4863		3086
No. non hydrogen	5523	5965		5602
No. of Mg^{2+}/Mn^{2+}	5/0	2/2		8/0
No. of ligand atoms	19	76		38
Solvent	160	346		222
R_{work}/R_{free} (%)[e]	17.6/22.2	15.9/19.4		19.9/24.1
r.m.s.d. bond (Å) / (angle)[f]	0.002/1.1	0.003/1.2		0.002/1.2
B-factor ($Å^2$)				
- Protein	43.3	31.3		36.7
- Ligand/Ion	41.8	40.9		37.6
- Solvent	44.1	38.9		39.6
Ramachandran Plot (%)[g]	98.6/1.4/0	98.0/2.0/0		98.6/1.4/0
PDB accession code	**8A70**	**8A73**		**8A74**

[a] Asymmetric unit
[b] The values in parentheses for resolution range, completeness, R_{merge} and I/σ (I) correspond to the highest resolution shell
[c] Data reduction was carried out with XDS and from a single crystal. Friedel pairs were treated as identical (or individual[*]) reflections
[d] $R_{merge}(I) = \Sigma_{hkl}\Sigma_j \mid I(hkl)_j - \langle I(hkl)\rangle \mid / \Sigma_{hkl} \Sigma_j I(hkl)_j$, where $I(hkl)_j$ is the jth measurement of the intensity of reflection hkl and <I(hkl)> is the average intensity
[e] $R = \Sigma_{hkl} \mid |F_{obs}| - |F_{calc}| \mid / \Sigma_{hkl} |Fobs|$, where R_{free} is calculated without a sigma cut off for a randomly chosen 5% of reflections, which were not used for structure refinement, and R_{work} is calculated for the remaining reflections
[f] Deviations from ideal bond lengths/angles
[g] Percentage of residues in favoured region / allowed region / outlier region

	*Pc*IDS1_F315A$_{Mg/Mn}$ GPP:PP	*Pc*IDS1$_{Mg}$ 3-Br-GPP	*Pc*IDS1_D319N$_{Mg}$ 3-Br-GPP	*Pc*IDS1$_{Mg}$ ZOL:IPP
**Crystal parameters**				
Space group	$P2_1$	$P2_1$	$P2_1$	$P2_1$
Cell constants	a=59.1 Å	a=59.5 Å	a=59.3 Å	a=58.0 Å
	b=78.8 Å	b=78.3 Å	b=78.0 Å	b=71.0 Å
	c=88.5 Å	c=88.9 Å	c=88.7 Å	c=94.1 Å
	β=102.2°	β=101.5°	β=101.3°	β=91.6°
Subunits / AU[a]	2	2	2	2
**Data collection**				
Beam line	X06SA, SLS	X06SA, SLS	X06SA, SLS	X06SA, SLS
Wavelength (Å)	1.0	0.92	0.92	1.0
Resolution range (Å)[b]	30-1.6	30-1.6	30-1.65	30-1.2
	(1.7-1.6)	(1.7-1.6)	(1.75-1.65)	(1.3-1.2)
No. observations	307971	515622	546209	817710
No. unique reflections[c]	98744	201678[*]	184588[*]	234153
Completeness (%)[b]	94.5/92.5	97.3/97.5	98.4/99.1	98.5/98.0
R_{merge} (%)[b, d]	5.0/54.2	4.7/52.7	4.1/52.2	7.0/46.3
I/σ (I)[b]	11.4/2.0	9.7/2.0	13.2/2.1	7.8/2.0
**Refinement (REFMAC5)**				
Resolution range (Å)	15-1.6	15-1.6	15-1.65	15-1.2
No. refl. working set	93694	98531	89533	222321
No. refl. test set	5747	5186	4712	11701
No. non hydrogen	5747	5908	5742	6476
No. of Mg^{2+}/Mn^{2+}	3/1	5/0	5/0	6/0
No. of ligand atoms	28	19	19	60
Solvent	354	513	312	731
R_{work}/R_{free} (%)[e]	14.8/18.9	19.2/15.8	15.5/17.5	12.8/15.3
r.m.s.d. bond (Å) / (angle)[f]	0.006/1.4	0.003/1.2	0.003/1.2	0.014/1.8
B-factor (Å^2)				
- Protein	31.3	32.2	29.5	19.0
- Ligand/Ion	40.8	29.9	32.4	11.6
- Solvent	38.9	45.8	36.9	29.3
Ramachandran Plot (%)[g]	98.1/1.9/0	98.0/2.0/0	98.4/1.6/0	98.5/1.5/0
PDB accession code	**8A78**	**8A7A**	**8A7B**	**8A7C**

[a] Asymmetric unit

[b] The values in parentheses for resolution range, completeness, R_{merge} and I/σ (I) correspond to the highest resolution shell

[c] Data reduction was carried out with XDS and from a single crystal. Friedel pairs were treated as identical (or individual[*]) reflections

[d] $R_{merge}(I) = \Sigma_{hkl}\Sigma_j \mid I(hkl)_j - \langle I(hkl)\rangle \mid / \Sigma_{hkl} \Sigma_j I(hkl)_j$, where $I(hkl)_j$ is the jth measurement of the intensity of reflection hkl and $\langle I(hkl)\rangle$ is the average intensity

[e] $R = \Sigma_{hkl} \mid |F_{obs}| - |F_{calc}| \mid / \Sigma_{hkl} |Fobs|$, where R_{free} is calculated without a sigma cut off for a randomly chosen 5% of reflections, which were not used for structure refinement, and R_{work} is calculated for the remaining reflections

[f] Deviations from ideal bond lengths/angles

[g] Percentage of residues in favoured region / allowed region / outlier region

	***Pc*IDS1$_{Mn}$ ZOL:IPP**	***Pc*IDS1$_{Mn}$ ZOL:IPP ano**	***Pc*IDS1$_{Mn (low)}$ ZOL:IPP**	***Pc*IDS1$_{Mn (low)}$ ZOL:IPP ano**
**Crystal parameters**				
Space group	$P2_1$	$P2_1$	$P2_1$	$P2_1$
Cell constants	a=57.8 Å<br>b=70.6 Å<br>c=94.1 Å<br>β=91.4°	a=57.8 Å<br>b=70.6 Å<br>c=94.1 Å<br>β=91.4°	a=58.1 Å<br>b=70.5 Å<br>c=94.9 Å<br>β=91.3°	a=58.1 Å<br>b=70.5 Å<br>c=94.9 Å<br>β=91.3°
Subunits / AU[a]	2	2	2	2
**Data collection**				
Beam line	X06SA, SLS	X06SA, SLS	X06SA, SLS	X06SA, SLS
Wavelength (Å)	1.0	1.89	1.0	1.89
Resolution range (Å)[b]	30-1.2<br>(1.3-1.2)	30-2.2<br>(2.3-2.2)	30-1.3<br>(1.4-1.3)	30-2.2<br>(2.3-2.2)
No. observations	735866	232317	559331	246713
No. unique reflections[c]	229848	73237[*]	180288	72747[*]
Completeness (%)[b]	97.5/97.6	96.9/94.9	96.0/94.4	95.2/92.1
R_{merge} (%)[b, d]	3.6/45.4	4.5/11.7	4.3/55.2	5.4/14.6
I/σ (I)[b]	13.8/2.1	18.0/7.6	12.1/2.1	5.9/15.5
**Refinement (REFMAC5)**				
Resolution range (Å)	15-1.2		15-1.3	
No. refl. working set	218246		171154	
No. refl. test set	11487		9008	
No. non hydrogen	6508		6325	
No. of Mg^{2+}/Mn^{2+}	0/6		6/6	
No. of ligand atoms	60		60	
Solvent	742		524	
R_{work}/R_{free} (%)[e]	13.2/15.2		14.2/16.8	
r.m.s.d. bond (Å) / (angle)[f]	0.011/1.7		0.018/2.1	
B-factor ($Å^2$)				
- Protein	18.4		20.3	
- Ligand/Ion	11.6		13.0	
- Solvent	28.7		27.7	
Ramachandran Plot (%)[g]	98.3/1.7/0		98.2/1.8/0	
PDB accession code	**8A7J**		**8A7K**	

[a] Asymmetric unit
[b] The values in parentheses for resolution range, completeness, R_{merge} and I/σ (I) correspond to the highest resolution shell
[c] Data reduction was carried out with XDS and from a single crystal. Friedel pairs were treated as identical (or individual[*]) reflections
[d] $R_{merge}(I) = \Sigma_{hkl}\Sigma_j \mid I(hkl)_j - \langle I(hkl)\rangle \mid / \Sigma_{hkl} \Sigma_j I(hkl)_j$, where $I(hkl)_j$ is the jth measurement of the intensity of reflection hkl and <I(hkl)> is the average intensity
[e] $R = \Sigma_{hkl} \mid |F_{obs}| - |F_{calc}| \mid / \Sigma_{hkl} |Fobs|$, where R_{free} is calculated without a sigma cut off for a randomly chosen 5% of reflections, which were not used for structure refinement, and R_{work} is calculated for the remaining reflections
[f] Deviations from ideal bond lengths/angles
[g] Percentage of residues in favoured region / allowed region / outlier region

	*Pc*IDS1$_{Mg}$ ZOL:GPP	*Pc*IDS1_I420A$_{Mg}$ GPP:MES	*Pc*IDS1_F315D$_{Mg}$ GPP:GOL
**Crystal parameters**			
Space group	$P3_221$	$P2_1$	$P2_1$
Cell constants	a=86.2 Å	a=59.0 Å	a=59.0 Å
	b=86.2 Å	b=78.4 Å	b=78.1 Å
	c=119.6 Å	c=88.0 Å	c=88.1 Å
		β=101.9°	β=101.4°
Subunits / AU[a]	1	2	2
**Data collection**			
Beam line	X06SA, SLS	X06SA, SLS	X06SA, SLS
Wavelength (Å)	1.0	1.0	1.0
Resolution range (Å)[b]	30-1.85	30-2.0	30-1.55
	(1.95-1.85)	(2.1-2.0)	(1.65-1.55)
No. observations	125376	144572	296804
No. unique reflections[c]	43158	51160	107827
Completeness (%)[b]	97.1/99.4	96.2/98.6	94.9/98.2
R_{merge} (%)[b, d]	6.0/57.9	9.9/55.1	3.9/50.9
I/σ (I)[b]	9.5/2.0	6.9/2.4	13.1/2.1
**Refinement (REFMAC5)**			
Resolution range (Å)	15-1.85	15-2.0	15-1.55
No. refl. working set	40913	48480	102435
No. refl. test set	2153	2551	5392
No. non hydrogen	3048	5484	5762
No. of Mg^{2+}/Mn^{2+}	3/0	4/0	5/0
No. of ligand atoms	19	31	25
Solvent	177	130	398
R_{work}/R_{free} (%)[e]	14.7/18.2	18.6/22.9	14.6/18.6
r.m.s.d. bond (Å) / (angle)[f]	0.004/1.2	0.002/1.2	0.013/1.8
B-factor ($Å^2$)			
- Protein	38.7	36.7	28.4
- Ligand/Ion	30.9	38.3	25.3
- Solvent	43.8	38.7	34.5
Ramachandran Plot (%)[g]	98.3/1.7/0	98.4/1.6/0	98.3/1.7/0
PDB accession code	**8A7L**	**8A7R**	**8A7U**

[a] Asymmetric unit

[b] The values in parentheses for resolution range, completeness, R_{merge} and I/σ (I) correspond to the highest resolution shell

[c] Data reduction was carried out with XDS and from a single crystal. Friedel pairs were treated as identical (or individual[*]) reflections

[d] $R_{merge}(I) = \Sigma_{hkl}\Sigma_j \mid I(hkl)_j - \langle I(hkl)\rangle \mid / \Sigma_{hkl} \Sigma_j I(hkl)_j$, where $I(hkl)_j$ is the jth measurement of the intensity of reflection hkl and <I(hkl)> is the average intensity

[e] $R = \Sigma_{hkl} \mid |F_{obs}| - |F_{calc}| \mid / \Sigma_{hkl} |Fobs|$, where R_{free} is calculated without a sigma cut off for a randomly chosen 5% of reflections, which were not used for structure refinement, and R_{work} is calculated for the remaining reflections

[f] Deviations from ideal bond lengths/angles

[g] Percentage of residues in favoured region / allowed region / outlier region

Supplementary Table 4. RMSD values between *Pc*IDS1 subunits in complex with different ligands and structurally related proteins.

Subunit 1	PDB ID[a]	Subunit 2	PDB ID	RMSD value [Å]	Nr. C_α in alignment
*Gg*FPPS[b]	1UBX	*Pc*IDS1$_{apo}$	8A6U_A	1.2	291
*Ec*FPPS[c]<br>DMSPP:IPP	1RQI_A	*Pc*IDS1$_{Mg}$<br>IPP:IPP	8A6V_A	1.8	195
*Hs*FPPS[d]<br>FPP	5JA0	*Pc*IDS1$_{Mn}$<br>GPP:GPP	8A73_A	1.0	300
*Pc*IDS1$_{apo}$	8A6U_A	*Pc*IDS1$_{apo}$	8A6U_B	0.6	314
*Pc*IDS1$_{Mg}$<br>IPP	8A6V_B	*Pc*IDS1$_{apo}$	8A6U_A	0.7	308
*Pc*IDS1$_{Mg}$<br>IPP:IPP	8A6V_A	*Pc*IDS1$_{Mg}$<br>IPP	8A6V_B	1.2	320
*Pc*IDS1$_{Mn}$<br>IPP:IPP	8A6Z_A	*Pc*IDS1$_{Mn}$<br>IPP:IPP	8A6Z_B	0.2	339
*Pc*IDS1$_{Mn}$<br>IPP:IPP	8A6Z_A	*Pc*IDS1$_{Mg}$<br>IPP:IPP	8A6V_A	0.2	342
*Pc*IDS1$_{Mg}$<br>GPP	8A70_A	*Pc*IDS1$_{Mg}$<br>IPP	8A6V_B	0.7	326
*Pc*IDS1$_{Mn}$<br>GPP:GPP	8A73_A	*Pc*IDS1$_{Mg}$<br>GPP	8A70_A	0.4	333
*Pc*IDS1_F315A$_{Mg}$<br>2GPP	8A74_A	*Pc*IDS1$_{Mg}$<br>GPP	8A70_B	0.2	334
*Pc*IDS1_F315A$_{Mg}$<br>2GPP	8A74_A	*Pc*IDS1_F315A$_{Mg}$<br>2GPP	8A74_B	0.4	314
*Pc*IDS1$_{Mg}$<br>3-Br-GPP	8A7A_A	*Pc*IDS1$_{Mg}$<br>GPP	8A70_A	0.2	334
*Pc*IDS1$_{Mg}$<br>ZOL:IPP	8A7C_A	*Pc*IDS1$_{Mg}$<br>IPP:IPP	8A6V_A	0.3	341
*Pc*IDS1$_{Mg}$<br>ZOL:IPP	8A7C_A	*Pc*IDS1$_{Mg}$<br>ZOL:IPP	8A7C_B	0.4	339
*Pc*IDS1$_{Mn}$<br>ZOL:IPP	8A7J_A	*Pc*IDS1$_{Mn}$<br>ZOL:IPP	8A7J_B	0.5	339
*Pc*IDS1$_{Mn}$<br>ZOL:IPP	8A7J_A	*Pc*IDS1$_{Mg}$<br>ZOL:IPP	8A7C_A	0.1	345
*Pc*IDS1$_{Mg}$<br>ZOL:GPP	8A7L	*Pc*IDS1$_{Mg}$<br>ZOL:IPP	8A7C_A	0.4	338

[a] Chain ID according to PDB deposition.
[b] *Gallus gallus*
[c] *Escherichia coli*
[d] *Homo sapiens*

References

1. D. J. Hosfield *et al.,* Structural basis for bisphosphonate-mediated inhibition of isoprenoid biosynthesis. *J. Biol. Chem.* **279**, 8526–8529 (2004), doi:10.1074/jbc.C300511200.
2. J. Park, M. Zielinski, A. Magder, Y. S. Tsantrizos, A. M. Berghuis, Human farnesyl pyrophosphate synthase is allosterically inhibited by its own product. *Nat. Commun.* **8**, 14132 (2017), doi:10.1038/ncomms14132.
3. D. Gritzalis *et al.,* Probing the molecular and structural elements of ligands binding to the active site versus an allosteric pocket of the human farnesyl pyrophosphate synthase. *Bioorg. Med. Chem. Lett.* **25**, 1117–1123 (2015), doi:10.1016/j.bmcl.2014.12.089.

Raw images for Supplementary Fig. 1.

a)

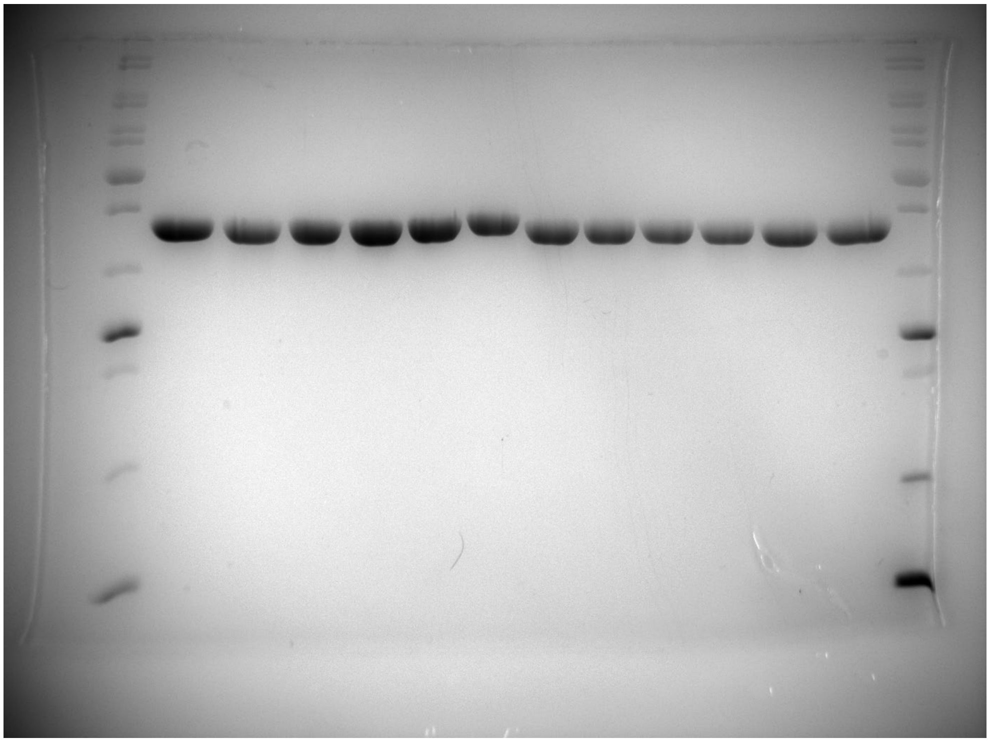

b)

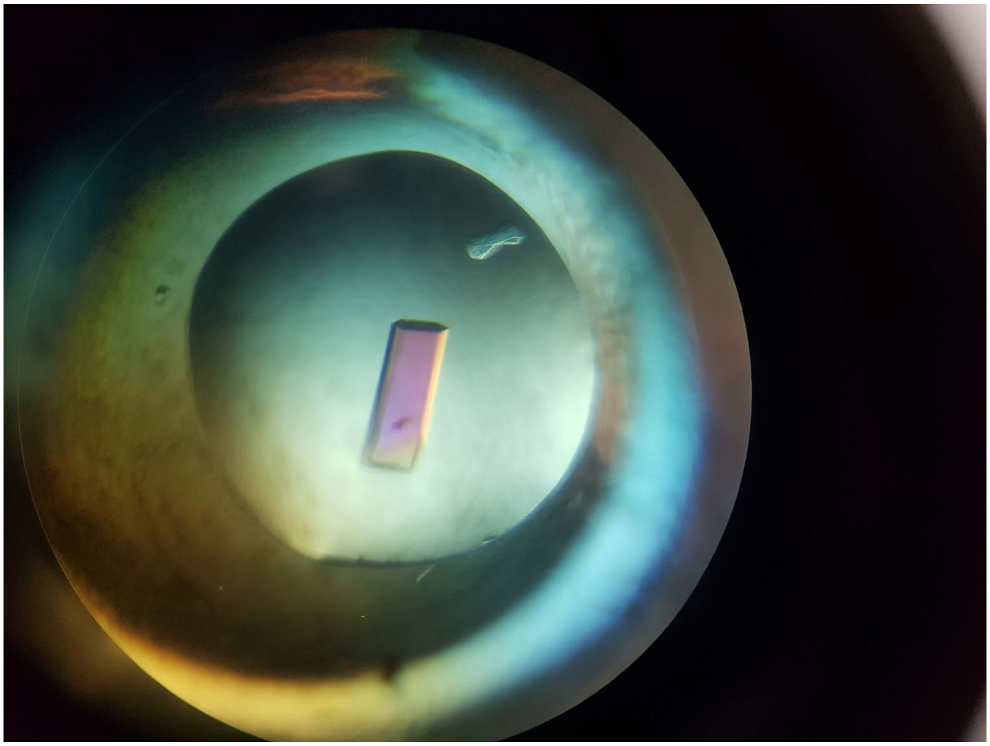

Supplement: Supplementary file 1 — Supplementary Figs. 1–11, Tables 1–4, references and additional source data. [file 41557_2023_1235_MOESM1_ESM.pdf]
